# Genome-wide association study identifies *ABCG1* as a susceptibility locus for tick-borne encephalitis

**DOI:** 10.1016/j.isci.2025.114017

**Published:** 2025-11-12

**Authors:** Piyush G. Gampawar, Manfred G. Sagmeister, Daniel Růžek, Nina A. Schweintzger, Edith Hofer, Benno Kohlmaier, Vendula Švendová, Petra Bogovič, Joanna M. Zajkowska, Lenka Krbková, Věra Štruncová, Auksė Mickienė, Daniela S. Kohlfürst, Astrid Sonnleitner, Andrea Fořtová, Michaela Berankova, Martina Pychova, Dace Zavadska, Neneh Sallah, Alexander Pichler, Dalibor Sedláček, Aleš Chrdle, Christoph Haudum, Barbara Obermayer-Pietsch, Per Hoffmann, Markus M. Nöthen, Mari-Liis Tammesoo, Andres Metspalu, Petr Husa, Karin Stiasny, Alexander Binder, Andrea Berghold, Franc Strle, Martin L. Hibberd, Werner Zenz, the EU-TICK-BO study group

**Affiliations:** 1 Department of Pediatrics and Adolescent Medicine, Division of General Pediatrics, Medical University of Graz, Graz, Austria; 2 Institute of Parasitology, Biology Centre of the Czech Academy of Sciences, České Budějovice, Czech Republic; 3 Infectious Diseases and Prevention Medicine Department, Veterinary Research Institute, Brno, Czech Republic; 4 Faculty of Science, Masaryk University, Brno, Czech Republic; 5 Core Facility Molecular Biology, Center for Medical Research, Medical University of Graz, Graz, Austria; 6 Institute for Medical Informatics, Statistics and Documentation, Medical University of Graz, Graz, Austria; 7 Department of Neurology, Medical University of Graz, Graz, Austria; 8 Department of Infectious Diseases, University Medical Center Ljubljana, Ljubljana, Slovenia; 9 Department of Infectious Diseases and Neuroinfections, Medical University of Białystok, Białystok, Poland; 10 Department of Children’s Infectious Diseases, Faculty of Medicine and University Hospital, Brno, Czech Republic; 11 Department of Infectious Diseases and Travellers Medicine, Medical Faculty Plzeň, Charles University, Prague, Czech Republic; 12 Department of Infectious Diseases, Lithuanian University of Health Sciences, Kaunas, Lithuania; 13 Department of Infectious Diseases, Faculty of Medicine, Masaryk University Brno and University Hospital Brno, Brno, Czech Republic; 14 Department of Paediatrics, Children’s Clinical University Hospital, Rīgas Stradiņa Universitāte, Riga, Latvia; 15 Department of Infection Biology, Faculty of Infectious and Tropical Diseases, London School of Hygiene and Tropical Medicine, London, UK; 16 Hospital Ceske Budejovice, Ceske Budejovice, Czech Republic; 17 Royal Liverpool University Hospital, Liverpool, UK; 18 Department of Internal Medicine, Division of Endocrinology and Diabetology, Medical University of Graz, Graz, Austria; 19 Institute of Human Genetics, University of Bonn, Bonn, Germany; 20 Estonian Genome Centre, Institute of Genomics, University of Tartu, Tartu, Estonia; 21 Center for Virology, Medical University of Vienna, Wien, Austria; 22 GSK, Stevenage, UK

**Keywords:** Genetics, Microbiology, Bioinformatics

## Abstract

Tick-borne encephalitis (TBE) is a viral infection of the central nervous system, caused by the tick-borne encephalitis virus (TBEV) presenting clinically as meningitis, meningoencephalitis, and meningoencephalomyelitis. To investigate genetic susceptibility to TBE, and its severe forms, we conducted a genome-wide association study in the European population comprising 1,600 TBE cases and 9,699 controls. We identified several suggestive (*p* < 1 × 10^−5^) intronic and exonic variants in *ABCG1*, the only gene significantly associated with TBE susceptibility. These variants were shown to influence *ABCG1* expression in peripheral blood, a finding corroborated by RNA expression analysis. *In vitro* inhibition or silencing of *ABCG1* significantly reduced TBEV replication in both neuronal cells and macrophages, highlighting the potential role of *ABCG1* in TBEV biology. Additionally, we detected a genome-wide significant variant within *TEX41*, located downstream of *ZEB1*, associated with severe forms of TBE. These findings provide novel insights into the genetic factors underlying TBE susceptibility and severity.

## Highlights

• Genome-wide association study implicates *ABCG1* in host susceptibility to TBE
• *ABCG1* variants modulate gene expression in peripheral blood
• Inhibition or silencing of *ABCG1* reduces TBEV titers in neuronal cells and macrophages
• Findings highlight the role of *ABCG1* in TBEV biology

## Graphical abstract

**Figure:**
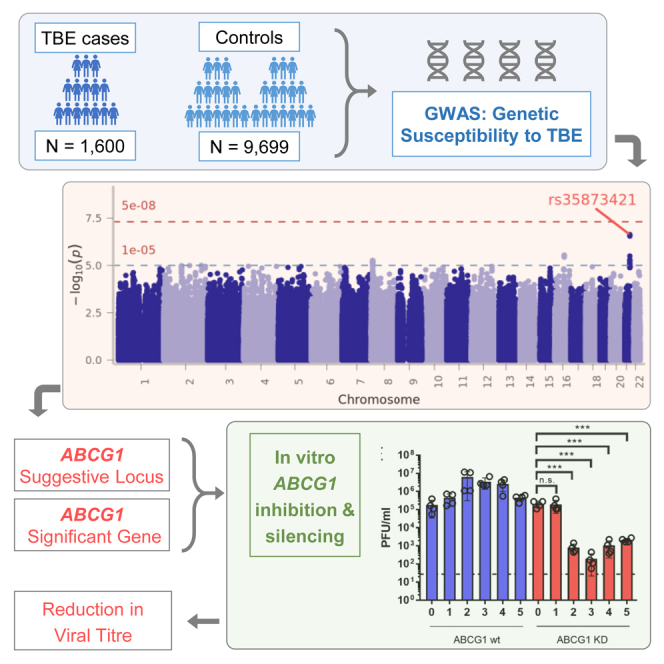

## Introduction

Tick-borne encephalitis (TBE) is a leading cause of viral infection of the central nervous system (CNS) in Europe and large parts of Asia, presenting a significant public health challenge.^1^^,^^2^ The causative agent, TBE virus (TBEV), is a neurotropic, single-stranded RNA virus from the *Orthoflavivirus* genus of the *Flaviviridae* family.^3^ TBEV shows substantial genetic diversity and is classified into three main subtypes, namely the European (TBEV-Eu), transmitted primarily by *Ixodes ricinus*, and the Siberian (TBEV-Sib), and Far Eastern (TBEV-FE), both vectored by *Ixodes persulcatus*.^4^^,^^5^

Clinically, infections caused by TBEV-Eu often follow a biphasic course, beginning with a febrile illness, an asymptomatic phase, and subsequent CNS involvement, including meningitis, encephalitis, myelitis or combinations thereof.^5^^,^^6^^,^^7^ Infections due to TBE-Eu has a case-fatality ratio (CFR) of usually 0.5%–2%, although severe neurological sequelae occur in up to 10%.^5^^,^^8^ Recent epidemiological data documented CFR of 0% · 4% and severe long-term sequelae in 5% · 4% of cases, alongside a significant increase in TBE incidence.^2^ The European Genetics Study of TBE (EU-TICK-BO), which includes a substantial proportion of patients from the current study, reported long-lasting sequelae in 16%–50% of patients.^6^

Infections caused by TBEV-FE are often more severe, frequently monophasic without an asymptomatic interval, and have higher CFRs (∼20%–40%) in reported neurologic cases.^5^^,^^8^ TBEV-Sib infections are generally intermediate, with CFR reported from ∼1% to 8% and occasional reports of chronic courses.^5^^,^^8^

TBE in children is generally characterized by a milder clinical course and favourable outcomes, yet, severe manifestations can occur.^9^^,^^10^ Although highly efficacious and safe vaccines against TBE are available and recommended for people living in or visiting high endemic areas,^11^ the vaccination coverage remains low in number of affected regions, and there is no specific treatment yet.^5^

Increasing evidence highlights the role of host genetic variation in susceptibility and response to infectious diseases, with genome-wide association studies (GWASs) offering valuable insights.^12^^,^^13^^,^^14^ While no GWAS has explored genetic susceptibility to TBE, candidate gene studies have been conducted but were limited by small sample sizes and inconsistent replication.^15^^,^^16^^,^^17^^,^^18^^,^^19^^,^^20^^,^^21^ GWAS of severe dengue, caused by another orthoflavivirus, identified susceptibility loci, emphasizing the role of host genetics in orthoflavivirus infections.^22^

To address the limited understanding of host genetic factors influencing susceptibility to TBE, we conducted a GWAS comprising 1,600 TBE cases from seven European countries with high TBE prevalence and 9,699 population-based controls. As a secondary analysis, we investigated genetic factors linked to the severe clinical manifestations of TBE, specifically encephalitis and myelitis. Finally, we performed targeted *in vitro* inhibition and silencing experiments in neuronal cells and macrophages, respectively, to test whether genes prioritized by the GWAS plausibly modulate TBEV biology, providing orthogonal functional context for the genetic findings.

## Results

### Study cohorts and clinical characteristics

#### European genetics study of tick-borne encephalitis cohort

**Figure 1:**
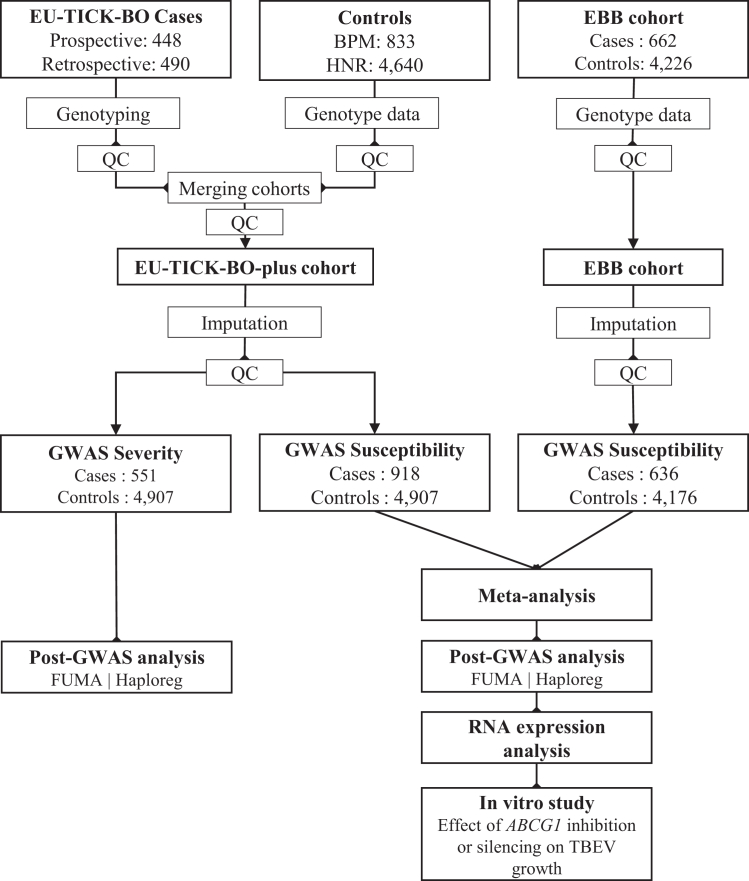
Workflow of the study TBE cases recruited within the EU-TICK-BO were genotyped. Genotype data were available from population-based BPM and HNR controls. All three cohorts underwent separate QC before being merged to form the EU-TICK-BO-plus cohort. The EBB cohort also underwent separate QC. Both the EU-TICK-BO-plus and EBB cohorts were imputed to the HRC reference panel, followed by a final QC step. Susceptibility to TBE was tested separately in the EU-TICK-BO-plus and EBB cohorts using a linear mixed model, followed by meta-analysis. Severity analysis included susceptibility to severe forms of TBE, including meningoencephalitis and meningoencephalomyelitis, and was performed only in the EU-TICK-BO-plus cohort. TBE, Tick-borne Encephalitis; EU-TICK-BO, European Genetics Study of Tick-borne Encephalitis; EBB, Estonian Biobank cohort; BPM, Austrian Biomarkers of Personalized Medicine study; HNR, German Heinz Nixdorf Recall Study; QC, Quality control; GWAS, Genome-Wide Association Study; HRC, Haplotype Reference Consortium.

A total of 938 TBE cases were recruited from hospitals across Austria, Czechia, Lithuania, Latvia, Poland, and Slovenia (Table S1). Of these, 355 (37.8%) had meningitis, 500 (53.3%) had meningoencephalitis, 63 (6.7%) had meningoencephalomyelitis, and 20 were unclassified. The mean age was 49.5 ± 17.4 years (range: 0.9–88.4), including 42 children (<18 years; mean:10.7 ± 5.0; range: 0.9–17.7) of which 57.6% were male. Population-based European ancestry controls were drawn from the German Heinz Nixdorf Recall Study (HNR) (*N* = 4,640)^23^ and the Austrian Biomarkers of Personalized Medicine study (BPM) (*N* = 833).^24^ Henceforth, we refer to the combination of EU-TICK-BO cases and controls as the EU-TICK-BO-plus cohort that comprised 918 cases and 4,907 controls after quality control (QC) (Figure 1).

#### Estonian Biobank (EBB) cohort

The EBB cohort^25^ included 662 TBE cases of which 32.6% were male. Detailed clinical diagnosis of TBE forms was unavailable for this cohort. Controls were EBB participants without a diagnosis of TBE or other viral meningitis or encephalitis. After QC, 636 cases and 4,176 controls were available for the analysis (Figure 1).

### Genome-wide association analysis of genetic susceptibility to TBE

In the discovery phase, 5,825 individuals from the EU-TICK-BO*-*plus cohort were analyzed using a generalized linear mixed model.^26^ After imputation and QC 7,007,551 SNPs were retained for analysis. Principal-component analysis (PCA) revealed overlap between cases and controls (Figure S1; S2; S3). After adjusting for sex and the first five PCs, we found minimal evidence of genomic inflation (λgc = 1.034) (Figure S4). Although no SNPs reached genome-wide significance, 54 variants in six loci were associated with TBE susceptibility at the suggestive significance (*p* < 1 × 10^−5^) (Figure S4). These loci could not be replicated in the EBB cohort (4,812 participants, 7,247,027 SNPs) (Figures S5–S7).

**Figure 2:**
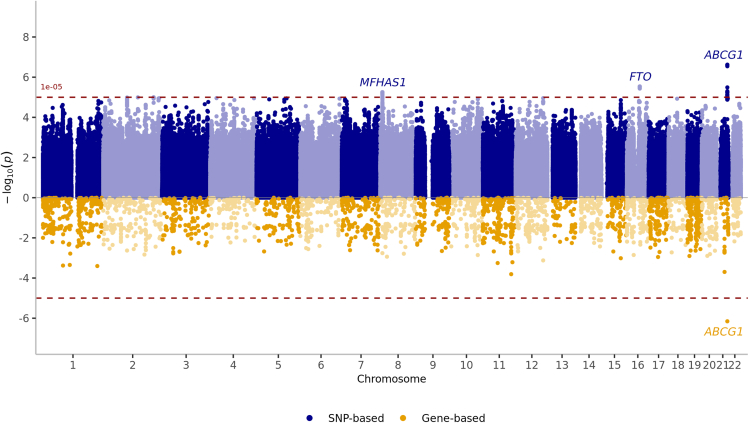
Miami plot illustrating the GWAS results for genetic susceptibility to TBE The top panel shows genotype-based associations, with loci surpassing the suggestive threshold (*p* < 1 × 10^−5^) annotated in blue. The bottom panel depicts gene-wide association results, highlighting genes exceeding the gene-wide significance threshold (*p* < 2 · 68×10^−6^) in yellow.

Subsequently, we performed a fixed-effects, inverse-variance meta-analysis combining the results of the EU-TICK-BO*-*plus and the EBB cohorts. There was no evidence of genomic inflation (λgc = 1.019) (Figure S8). None of the variants reached genome-wide significance, although, there were 52 genome-wide suggestive associations (*p* < 1 × 10^−5^) clustered into three loci on chromosomes 8, 16, and 21 with the chromosome 21 SNP showing the highest significance (Table 1; Figure 2). Using LD (linkage disequilibrium)-based clumping, we identified two independent SNPs within the chromosome 21 locus. The lead SNP, rs35873421, was located in the fifth intron of the ATP binding cassette subfamily G member 1 (*ABCG1)* (Minor Allele Frequency (MAF) = 0.23, odd’s ratio (OR) = 1.35, *p* = 2.39 × 10^−7^). The second SNP, rs3787986, was located in the first intron of *ABCG1* (MAF = 0.15, OR = 1.38, *p* = 3.2 × 10^−6^). Additionally, this locus contained four exonic and 12 intronic SNPs (Table S2; Figure S9). Our sample size had ∼80% power to detect OR≥1.33 at MAF = 0.23 (two-sided α = 5 × 10^−8^) and the lead locus (OR = 1.35 at MAF = 0.23) corresponds to ∼88% power.

**Table 1:** Meta-analysis of GWAS of susceptibility to TBE

SNP	Chr	Pos	Ref	Alt	Nearest Gene	EU-TICK-BO*-*plus				EBB				Meta-Analysis	
						OR	95%CI	MAF	P	OR	95%CI	MAF	P	OR	P
rs35873421	21	43694730	G	A	*ABCG1*	1.29	1.07, 1.56	23%	7.39×10^−3^	1.38	1.20, 1.58	24%	8.74×10^−6^	1.35	2.39×10^−7^
rs3787986	21	43678808	C	T	*ABCG1*	1.25	1.00, 1.56	15%	5.44×10^−2^	1.46	1.30, 1.72	15%	1.23×10^−5^	1.38	3.23×10^−6^
rs1016399	16	54185626	T	G	*FTO*	1.23	1.04, 1.45	36%	1.57×10^−2^	1.30	1.14, 1.47	34%	5.40×10^−5^	1.27	2.88×10^−6^
rs76620708	8	8619083	C	T	*MFHAS1*	0.70	0.57, 0.86	16%	7.95×10^−4^	0.78	0.67, 0.91	17%	1.61×10^−3^	0.75	5.58×10^−6^

EU-TICK-BO-plus, European Genetics Study of Tick-borne Encephalitis plus cohort; EBB, Estonian Biobank cohort; SNP, Single Nucleotide Polymorphism; Chr, Chromosome; Pos, Position; Ref, Reference Allele; Alt, Alternate allele; OR, Odd’s Ratio; MAF, Minor Allele Frequency; 95% CI, 95% Confidence Interval; ABCG1, ATP binding cassette subfamily G member; 1FTO, FTO alpha-ketoglutarate dependent dioxygenase; MFHAS1, multifunctional ROCO family signaling regulator 1.

The second locus on chromosome 16 contained six suggestive SNPs (*p* < 1 × 10^−5^), with the top hit being rs1016399 (MAF = 0.34, OR = 1.27, *p* = 2.88 × 10^−6^), located downstream of the FTO alpha-ketoglutarate dependent dioxygenase (*FTO)* gene. The third locus on chromosome 8 had 29 suggestive SNPs (*p* < 1 × 10^−5^) upstream of the multifunctional ROCO family signaling regulator 1 (*MFHAS1)* gene, with rs76620708 as the top SNP (MAF = 0.17, OR = 0.75, *p* = 5.58 × 10^−6^), (Table 1).

Gene-based association analysis, performed using MAGMA,^27^ revealed that *ABCG1* was the only gene to reach gene-wide significance (*p* = 2.64 × 10^−6^) after correcting for multiple testing across 18,917 protein-coding genes (Figure 2).

### TBE associated GWAS variants modulate *ABCG1* expression in blood and immune cells

We used FUMA^28^ to annotate GWAS results and further explored the blood expression quantitative trait loci (eQTL) database from the eQTLGen consortium.^29^ The lead SNP, rs35873421, showed a nominally significant association with *ABCG1* expression in peripheral blood, while the second independent SNP, rs3787986, exhibited a strong association, with the alternate T allele linked to higher *ABCG1* expression (*Z* score = +5.7).^29^ Additionally, 11 suggestive SNPs (*p* < 1 × 10^−5^) in high LD (r^2^ > 0.9) with rs3787986 were also associated with increased *ABCG1* expression (Table S3). Importantly, these eQTLs were statistically significant, with FDR-adjusted *p* < 0.001.

Analysis using methylation QTL (meQTL) data from deCODE genetics^30^ revealed that rs35873421 is associated with decreased CpG methylation within a 12 bp region in *ABCG1*. The A allele reduces methylation levels by 0.145 standard deviations per allele, indicating its role as an allele-specific meQTL.

Functional annotation through HaploReg^31^ showed that rs35873421 overlaps with a 6_EnhG region marked by H3K4me1 in primary hematopoietic stem cells, indicating a role in modulating gene expression through enhancer activity. Additionally, rs3787986 is located within enhancer regions of immune-related cells (B and T cells) and brain tissues, marked by H3K4me1 and other strong enhancer markers (H3K27ac, H3K9ac). These enhancer elements suggest that rs3787986 may regulate *ABCG1* expression in immune cells and brain tissue, potentially affecting the gene function.

### rs3787986 influences *ABCG1* mRNA expression in peripheral blood

Using 23 samples from the EU-TICK-BO cohort with RNA sequencing data, we implemented a generalized linear model adjusted for sex to assess the differences in *ABCG1* gene expression between people with and without the rs3787986 variant. We found a significant increase in *ABCG1* expression in heterozygous compared to homozygous individuals (*p* < 0.05), reinforcing the role of rs3787986 as an eQTL (Figure S10).

### *In vitro* inhibition or silencing of *ABCG1* decreases TBEV growth

**Figure 3:**
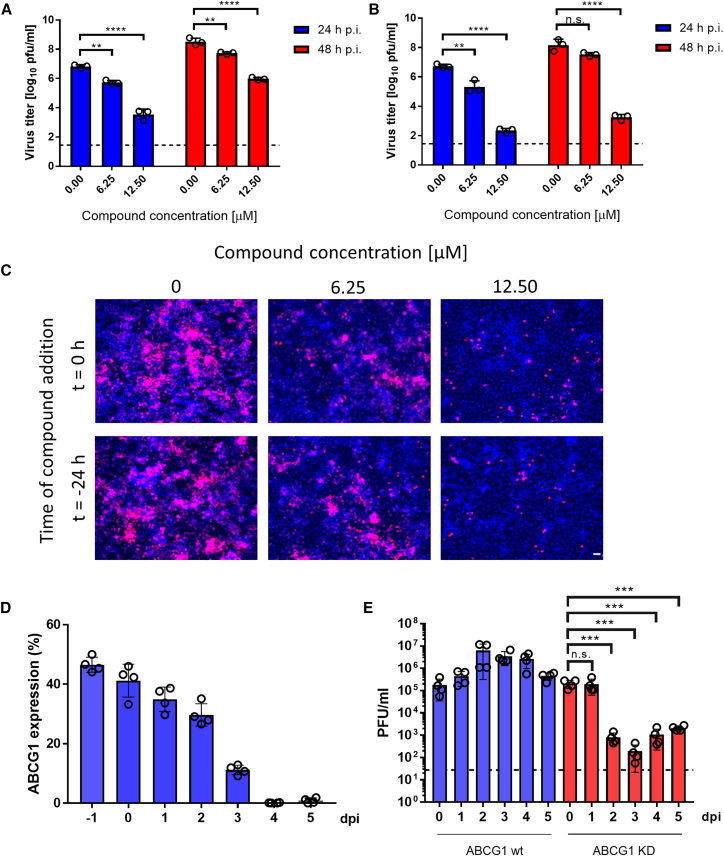
Effect of ABCG1 inhibition on TBEV growth (A–E) Neuronal UKF-NB-4 cells were infected with TBEV at a MOI of 0.1 and treated with different concentrations of the ABCG1 inhibitor benzamil, either simultaneously with infection (*N* = 3) (A) or 24 h before infection (B). Supernatant medium was collected at 24 h or 48 h post infection (p.i.), and viral titer was determined using a plaque assay. The data are expressed as the mean ± SE. The dashed line indicates the detection limit of the assay (A and B). Confluent monolayers of human neuroblastoma cells (UKF-NB-4) were infected with recombinant TBEV expressing the mCherry reporter gene. Cells were treated with 0, 6.25, or 12.5 μM benzamil either at the time of infection or 24 h before. The mCherry expression was examined by fluorescence microscopy 48 h after infection. Cell nuclei were visualized with Hoechst 33342 dye. Scale bar, 40 μm (C). Expression of ABCG1 was silenced in RAW 264.7 macrophage cells using RNAi (*N* = 4), initiated 2 days before infection (day −2 dpi). The efficiency of *ABCG1* downregulation was assessed by RT-qPCR every 24 h for up to 7 days post-transfection, with relative expression levels compared to controls shown (D). RAW 264.7 cells with silenced *ABCG1* expression (*N* = 4) and control cells (*N* = 4) treated with negative control siRNA were infected with TBEV at a MOI of 0.1. The supernatant medium was collected daily from day 0–5 dpi, and viral titers were determined using a plaque assay. The results are presented as the mean ± SE in D and E. n.s. not significant ∗*p* ≤ 0.05; ∗∗∗*p* ≤ 0.001; ∗∗∗∗*p* ≤ 0.0001. The analysis was performed using the Kruskal-Wallis test, followed by Dunn’s multiple comparison test.

Experiments employed the TBEV-Hypr strain (European subtype). TBEV-infected neuroblastoma cells (UKF-NB-4) were treated with the *ABCG1* inhibitor^32^ benzamil at concentrations of 0, 6.25, and 12.5 μM, both 24 h prior to infection (pre-treated) and simultaneously with TBEV infection (*N* = 3). Viral load quantification at 24- and 48-h post-infection revealed a significant ∼3-fold reduction (*p* < 0.0001) in the simultaneously treated group with 12.5 μM benzamil after 24 h and ∼2-fold reduction (*p* < 0.0001) after 48 h (Figure 3A). In the pre-treated group, viral titers dropped ∼4-fold (*p* < 0.0001) after 24 h and ∼5-fold (*p* < 0.0001) after 48 h compared to the untreated control group (Figure 3B). Moreover, the 48-h post-infection comparison between pre-treated and simultaneously treated groups exposed to 12.5 μM benzamil demonstrated a roughly half reduction in viral load in the pre-treated group (Figure 3B) compared to the simultaneously treated group (Figure 3A), as confirmed by immunostaining (Figure 3C).

We employed siRNA to silence *ABCG1* expression in macrophage cell line (*N* = 4). Treatment with *ABCG1*-specific siRNA reduced relative *ABCG1* expression compared to negative controls by approximately 50% on day 1 post-application and by 90% on day 5 post-application. By days 5–7, the relative expression of *ABCG1* was virtually undetectable (Figure 3D). Two days post-siRNA treatment (0 dpi in Figures 3D and 3E), the cells were infected with TBEV. Viral titers in the culture supernatants were monitored daily over a 5-day period (Figure 3E). In control cells treated with negative control siRNA, viral titers increased substantially between day 0 and day 2, demonstrating active viral replication. This was followed by a slight decline in titers between days 3 and 5, though levels remained higher than those observed on day 0. In contrast, viral titers in cells treated with *ABCG1*-specific siRNA dropped significantly between days 2 and 5 post-infection (*p* < 0.001) (Figure 3E).

In summary, *in vitro* inhibition or silencing of *ABCG1* led to a significant reduction in TBEV titers.

### GWAS of susceptibility to severe forms of TBE

**Figure 4:**
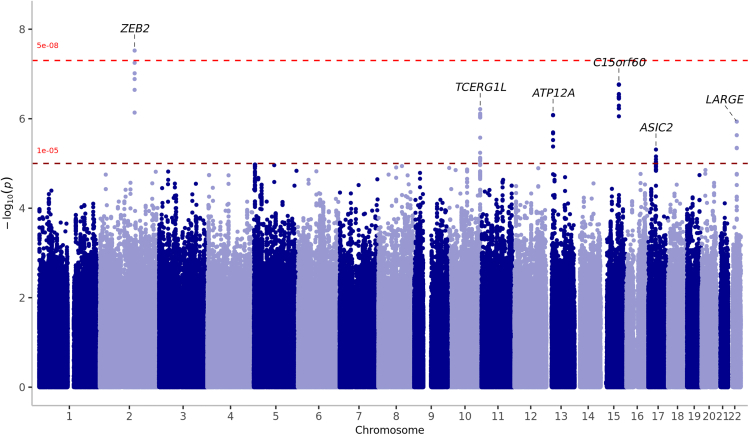
Manhattan plot displaying GWAS results for genetic susceptibility to severe forms of TBE Loci surpassing the suggestive threshold (*p* < 1 × 10^−5^) are annotated, with a significant association identified on chromosome 2 in the ZEB2 region, exceeding the genome-wide significance threshold (*p* < 5 × 10^−8^). Severe form of TBE includes meningoencephalitis and meningoencephalomyelitis.

To further investigate the role of host genetics in TBE severity, we conducted a GWAS comparing severe TBE cases (meningoencephalitis or meningoencephalomyelitis) with population-based controls within the EU-TICK-BO-plus cohort. As in the susceptibility analysis, models were adjusted for sex and the first five PCs. There was no evidence of systematic genomic inflation (λgc = 0.98) (Figure S11). Unlike the GWAS of all TBE outcomes, we found a genome-wide significant association with the SNP rs144051692 on chromosome 2 (MAF = 0.016, OR = 12.6, *p* = 3×0^−8^). The lead SNP was located in the second intron of the Testis Expressed 41 (*TEX41)* non-protein-coding gene, downstream of the zinc finger E-box binding homeobox 2 (*ZEB2)*. Furthermore, we identified 51 genome-wide suggestive associations (*p* < 1 × 10^−5^) on chromosome 10, 13, 15, 17, and 22 (Figures 4 and S12; Table S4). No gene reached gene-wide significance in the gene-based analysis. Functional annotation indicated that rs144051692 is located in an enhancer region within bone marrow-derived cultured mesenchymal stem cells.

## Discussion

We conducted the first GWAS on genetic susceptibility to TBE, recruiting participants across seven European countries with high TBE prevalence. We identified multiple intronic and exonic *ABCG1* variants showing suggestive significance (*p* < 1 × 10^−5^), with *ABCG1* emerging as the only gene significantly associated in the gene-based analysis. Two independent SNPs, rs35873421 and rs3787986, were identified as a meQTL and eQTL, respectively, influencing *ABCG1* expression in peripheral blood, a finding further supported by RNA expression analysis. *In vitro* inhibition or silencing of *ABCG1* significantly reduced TBEV titers in both neuronal cells and macrophages, highlighting the potential role of *ABCG1* in TBEV biology. Additionally, an intronic *TEX41* variant downstream of *ZEB1* was significantly associated with severe TBE.

In the meta-analysis, two independent SNPs, rs35873421 (MAF:23%, OR:1.35) and rs3787986 (MAF:15%, OR:1.38) were suggestively (*p* < 1 × 10^−5^) associated with TBE risk but did not meet the conventional genome-wide significance threshold (*p* < 5 × 10^−8^) (Table 1). Interestingly, both risk alleles had relatively lower OR (1.29 and 1.25) in the EU-TICK-BO-plus cohort than the EBB cohort (1.38 and 1.46), despite similar allele frequencies, suggesting cohort-specific factors influencing the association. A plausible explanation is the difference in disease prevalence between the cohorts. In the EU-TICK-BO-plus cohort, cases were recruited predominantly from countries with high TBE prevalence,^2^ whereas controls were recruited from low-risk regions of Germany^33^ and from Austria, a country with very high vaccination coverage.^34^ This disparity is likely to have attenuated the association observed in the EU-TICK-BO-plus cohort. Conversely, the EBB cohort, comprising people from Estonia, a region with higher TBE prevalence,^2^ may showed stronger associations due to greater viral exposure or increased susceptibility among carriers of these risk variants, potentially driven by unique genetic or environmental factors. Notably, the use of population-based common controls without accounting for pathogen exposure in GWAS of infectious diseases has been shown to bias effect estimates toward the null.^35^

Further analysis of rs35873421 showed its role as an allele-specific meQTL for a 12-bp region on chromosome 21 (GRCh38 location: chr21:42,269,247-42,269,259). Specifically, the reference allele G is associated with a CpG methylation rate of 63%, while the alternate allele T is associated with a reduced methylation rate of 59%, lowering CpG methylation by 0.145 standard deviations per allele (FDR *p* = 6.5 × 10^−14^).^30^ This region likely harbors or is proximal to regulatory elements affecting *ABCG1* expression through methylation changes, as suggested by clustered interactions of GeneHancer regulatory elements^36^ in UCSC genome browser.^37^

The second variant, rs3787986, is an eQTL for *ABCG1* expression in peripheral blood, supported by eQTLGen consortium data^29^ (over 31,000 samples across 37 cohorts).^29^ rs3787986 and 12 suggestive SNPs in high LD (r^2^ > 0.9) correlated with increased *ABCG1* expression (Table S3). This was further corroborated by RNA sequencing data, where individuals heterozygous for the T allele showed significantly higher *ABCG1* expression compared to those homozygous for the C allele. rs3787986 is enriched in epigenomic annotations in immune cells (monocytes, neutrophils, B cells, T cells) and brain regions, based on chromatin state models from histone modifications and DNase sensitivity data.^31^^,^^38^ These findings collectively support rs3787986 as a potential modulator of *ABCG1* expression specifically within peripheral blood, potentially contributing to immune cell-related regulatory processes.

Previous studies identified associations between *MICA* and *MICB* (MHC class I chain-related genes) within the extended major histocompatibility complex (MHC) region and hepatitis C^39^ and dengue virus^22^^,^^40^ infections, both members of the *Flaviviridae* family. In our analysis, the most significant SNP in the MHC region was rs113708600 (MAF = 0.96; OR = 1.53; *p* = 0.000495), located in the intronic region of *MICA*. This association, although notable, didn’t reach suggestive significance, likely due to limited statistical power. Larger studies are required to clarify the role of the MHC in TBE susceptibility.

Our GWAS findings are strongly supported by *in vitro* experiments. Pharmacological inhibition of *ABCG1* with benzamil significantly reduced TBEV titers in neuronal cells, while siRNA-mediated silencing of *ABCG1* in macrophages led to a marked decrease in viral replication. These results are particularly relevant, as macrophages are key mediators of early viral dissemination and immune modulation after TBEV entry, while neuronal cells, the primary targets in the brain, are central to the severe neurological complications of TBE.^34^ *ABCG1* plays a key role in lipid transport and homeostasis, modulating lipid rafts, which are critical for signal transduction and immune cell function.^41^ Lipid rafts also serve as platforms for viral entry and replication.

Our findings are consistent with previous research on Zika virus, another orthoflavivirus, where *ABCG1* upregulation was observed in infected retinal pigment epithelial cells. Inhibition of *ABCG1* with benzamil significantly reduced Zika viral infectivity, paralleling our results and further supporting a pro-viral role for *ABCG1* activity in orthoflavivirus infections.^32^ Additionally, the involvement of ABC transporter genes in TBEV infection is further highlighted by the association of *ABCC6* with survival outcomes in TBEV-infected mice.^42^ These findings underscore the broader relevance of ABC transporters in modulating viral pathogenesis and host immune responses.

Another important finding of our study is the identification of a significant association between an intronic *TEX41*variant, downstream of *ZEB2*, and susceptibility to severe TBE. *TEX41* is an RNA gene encoding a long noncoding RNA, previously associated with inflammatory bowel disease, coronary artery disease, and various cancers.^43^ *ZEB2* encodes a transcription factor essential for immune cell differentiation and neurodevelopment.^44^ The combination the low MAF, a comparatively large effect size, limited numbers of severe cases, and the multiple-testing burden, a false-positive association cannot be excluded, highlighting the need for replication in other cohorts.

Our study is the first GWAS on TBE susceptibility with substantially increased statistical power compared to previous smaller, candidate gene studies, marking a major advancement in the field. The use of clinically and laboratory validated diagnostic criteria enabled accurate phenotypic classification, particularly with regard to disease severity, thereby facilitating a focused investigation of genetic associations with severe TBE outcomes. We addressed potential confounding from population structure by analyzing central and northern European cohorts separately and adjusting for ancestry using PCs and generalized linear mixed models. Importantly, our findings provide a valuable foundation for future investigations into host genetic susceptibility to TBE, with potential implications for both preventive and therapeutic strategies.

In conclusion, our study provides novel insights into the genetic factors underlying TBE susceptibility and severity, representing a crucial initial step in understanding the genetic basis of TBE. These insights may inform future research and contribute to the development of targeted preventive and therapeutic approaches.

### Limitations of the study

Despite these strengths, several limitations must be acknowledged. First, the overall sample size remains modest by GWAS standards, and the lack of an independent replication cohort, together with and the unavailability of TBE cases in widely used genetic biobanks, constrain the generalizability of our findings. Moreover, GWAS was conducted in European cohorts and our *in vitro* experiments used a TBEV-Eu Hypr strain. Consequently, replication in TBEV-Sib/FE and diverse populations, alongside additional functional studies, is needed to assess generalizability. Second, cases and controls in the EU-TICK-BO-plus cohort were recruited across multiple countries and genotyped separately, introducing the risk of population stratification and technical artifacts. To mitigate this, we applied rigorous QC procedures, adjusted for PCs, and implemented genomic control and mixed-model approaches. Nevertheless, differences in TBEV exposure and vaccination status between cases and controls may have biased effect estimates. Third, clinical metadata, including comorbidities, were not consistently available across all sites, limiting our ability to account for potential clinical confounders. Although recruitment protocols were harmonized where possible, the multicentre nature of the study may have introduced participation bias or variation in case severity. Fourth, in the EBB cohort, exclusion of other viral meningitis and encephalitis cases relied on ICD-10 coding, which may carry a risk of diagnostic misclassification and limited clinical detail in this cohort also limited the scope of severity analyses. Finally, the functional assays provide supportive but preliminary evidence for a role of *ABCG1* in TBEV replication that needs further experimental validation using *in vivo* studies.

## Resource availability

### Lead contact

Information and requests for resources should be directed to the lead contact Dr. Werner Zenz (werner.zenz@medunigraz.at).

### Materials availability

The study did not generate new unique reagents.

### Data and code availability

• Data: The summary statistics of the GWAS have been deposited at Zenodo and are publicly available as of the date of publication at https://zenodo.org/records/17364606.
• Code: This paper does not report original code. The data are produced using publicly available tools that are listed in the key resources table.

## Acknowledgments

The Austrian node at the Medical University of Graz was financially supported by Land Steiermark (Office of the Regional Government of Styria, Department of Economy, Tourism, Science and Research, Austria; GZ: Abt.12 – 16.K-8/2012-20) and by Pfizer Corporation Austria GesmbH through an investigator-initiated research agreement (W1232345). DR was supported by the National Institute of Virology and Bacteriology project (Programme EXCELES, Project ID LX22NPO5103), funded by the European Union – Next Generation EU, and by the Czech Science Foundation (grant no. GA23-07160S).

Many thanks to all hospital staff of various professions from all participating centers for their caring and often challenging work in diagnosing and treating patients with tick-borne encephalitis. Members of the EU-TICK-BO STUDY GROUP (in alphabetical order) L. Bridina, Riga Stradins University, Latvia; Z.A. Litauniece, Department of Neurology and Neurosurgery, Riga East University Hospital, Riga, Latvia; J. Pakalniene, Lithuanian University of Health Sciences, Lithuania; P. Repata University Hospital Plzen, Czech Republic; D. Sedláček, University Hospital Plzen, Czech Republic; A. Siemieniako, Medical University Bialystok, Poland; S. Unuk, University Clinical Center Maribor, Slovenia.

## Author contributions

Conceptualization: P.G.G., M.G.S., D.R., and W.Z.; data curation: P.G.G., M.G.S., D.R., N.A.S., B.K., A.F., and M.B.; formal analysis: P.G.G., M.G.S., and N.S.; funding acquisition: W.Z.; investigation: P.G.G., M.G.S., D.R., A.F., and M.B.; methodology: P.G.G., M.G.S., D.R., N.A.S., A.F., and M.B.; resources: D.R., W.Z., P.B., J.M.Z., L.K., V.S., A.M., D.S.K., A.S., M.P., D.Z., A.P., D.S., A.C., C.H., B.O.-P., P.H., M.M.N., M.-L.T., A.M., P.H., K.S., and F.S.; supervision: E.H., N.A.S., M.L.H., and W.Z.; writing – original draft: P.G.G.; writing – review and editing: P.G.G., M.G.S., D.R., N.A.S., E.H., B.K., V.S., P.B., J.M.Z., L.K., V.S., A.M., D.S.K., M.P., D.Z., N.S., A.P., D.S., A.C., A.M., P.H., K.S., F.S., M.L.H., and W.Z.

## Declaration of interests

The authors declare no competing interests.

## STAR★Methods

### Key resources table

**Table:** 

REAGENT or RESOURCE	SOURCE	IDENTIFIER
**Bacterial and virus strains**		

TBEV Hypr strain	Collection of Arboviruses, Institute of Parasitology, Biology Centre of the Czech Academy of Sciences, Ceske Budejovice, Czech Republic	N/A

**Chemicals, peptides, and recombinant proteins**		

L-15-Medium (Leibovitz)	Sigma Aldrich	L5520
Iscove’s modified Dulbecco’s medium	Sigma Aldrich	I3390
Antibiotic/Antimycotic Solution	Capricorn Scientific	AAS-B/2
1% L-glutamine	Sigma Aldrich	59202C
FBS	Biosera	FB-1001/ 100
1% Glutamine	Biosera	MS0104101J
Silencer Select Pre-designed siRNA targeting *ABCG1*	Life Technologies	s61797
Silencer™ Select Negative Control No. 1 siRNA	Thermo Fisher Scientific	4390843
Lipofectamine™ RNAiMAX Transfection Reagent	Thermo Fisher Scientific	13778100
Opti-MEM™ Reduced Serum Medium	Gibco, Life Technologies	31985062
Benzamil hydrochloride hydrate (Benzamil_	Sigma Aldrich	B2417

**Critical commercial assays**		

Promega Maxwell® 16 LEV Blood DNA Kits	Promega Corporation	WI 53711
Quant-iT™ PicoGreen™ dsDNA Assay Kits	Invitrogen	P7589
Cell Counting Kit-8	Dojindo Molecular Technologies	CCK-8
QIAamp Viral RNA Mini Kit	QIAGEN	52904
QIAsymphony PAXgene Blood RNA Kit	QIAGEN	762635
NEBNext® Globin & rRNA Depletion Kit	New England Biolabs	E7750L
NEBNext® Ultra™ II Directional RNA Library Prep Kit for Illumina®	New England Biolabs	E7765

**Deposited data**		

Summary statistics	This paper	https://zenodo.org/records/17364606

**Experimental models: Cell lines**		

Porcine kidney stable (PS) cells	National Cell Culture Collection, National Institute of Public Health, Prague, Czech Republic	N/A
human neuroblastoma (UKF-NB-4) cells	Professor Tomáš Eckschlager, 2^nd^ School of Medicine, Charles University, Prague, Czech Republic	N/A
RAW 264.7	ATCC	TIB-71

**Software and algorithms**		

Plink and Plink2	Chang et al.^45^	https://www.cog-genomics.org/plink/2.0/
fastGWA-GLMM	Jiang et al.,^26^	https://yanglab.westlake.edu.cn/software/gcta/#fastGWA-GLMM
Michigan Imputation Server	Das et al.^46^	https://imputationserver.sph.umich.edu/
Bigsnpr	Prive et al.^47^	
STAR aligner	Dobin et al.^48^	https://github.com/alexdobin/STAR
featureCounts	Liao et al.^49^	https://subread.sourceforge.net/featureCounts.html
DESeq2	Love et al.^50^	https://bioconductor.org/packages/devel/bioc/vignettes/DESeq2/inst/doc/DESeq2.html
QC toolbox		https://www.chg.ox.ac.uk/∼wrayner/tools/

### Experimental model and study participant details

#### Study cohorts

##### European genetics study of tick-borne encephalitis cohort

For the study, TBE cases were recruited people both prospectively and retrospectively from the participating hospitals in Austria, Czech Republic, Latvia, Lithuania, Poland and Slovenia. Prospective recruitment was conducted across participating centres between 2014 and 2017. For diagnosis of TBE, clinical and laboratory criteria from the European Centre for Disease Prevention and Control were followed across the recruitment sites.^51^ Patients were included if they met clinical criteria for central nervous system inflammation (e.g., meningitis, meningoencephalitis, meningoencephalomyelitis) and at least one laboratory criterion: serum IgM and IgG antibodies, cerebrospinal fluid IgM antibodies, or seroconversion or a four-fold rise in TBE-specific IgG in paired serum samples We included only patients with confirmed TBE, meeting the clinical and laboratory criteria.^6^

Information on the TBEV subtype from these cases was not available. TBE cases were further clinically classified by severity into mild forms (meningitis only) and severe forms (meningoencephalitis and meningoencephalomyelitis).^6^

##### Estonian Biobank cohort

In EBB, TBE diagnosis was established either via self-reported history of TBE in the baseline DNA donor questionnaire or extracted from electronic health records using ICD-10 codes (A84, A84·1, A84·9)^25^

##### Population controls

We obtained ethnically matched controls of European ancestry from the German HNR cohort and the Austrian BPM cohort.^24^ History of TBE or vaccination against TBE was not available from population controls. For EBB, controls were participants without a diagnosis of TBE or other viral meningitis or encephalitis as determined by ICD-10 codes.

All subjects, and/or their parents in the case of minors, provided written informed consent for participation in genetic studies. The study protocols were approved by the following ethics committees: Medical University of Graz, Austria (25-554 ex 12.13; 24-224 ex 11/12); Ministry of Health of the Republic of Slovenia (178/02/13; 37/12/13); Lithuanian University of Health Sciences, Kaunas (BE-2-33); Medical University of Bialystok (R-I-002/586/2013); Riga Stradins University (E-9 (2)); Medical University of Vienna (1849/2016); University Hospital Plzen (7.11.2013); University Hospital Brno (20.11.2016); University Hospital Essen (99-69-1200); and the Estonian Biobank (1.1-12/624).

For the GWAS, we defined two cohorts based on the discovery and replication phases. The discovery cohort, referred to as the EU-TICK-BO*-*plus cohort, consisted of TBE cases from EU-TICK-BO and population controls from the HNR and BPM studies. In the replication phase, we used TBE cases and controls from the EBB.

#### Cell lines

Porcine kidney stable (PS) cells used for plaque assay were cultivated in Leibovitz (L-15; ref. number L5520; Sigma) medium at 37 °C (without CO_2_ supplementation), and human neuroblastoma (UKF-NB-4) cells were cultured in Iscove’s modified Dulbecco’s medium (IMDM; ref. number I3390; Sigma) at 37 °C and 5% CO_2_. PS were obtained from the National Cell Culture Collection, National Institute of Public Health, Prague, Czech Republic. UKF-NB-4 cells were kindly provided by Professor Tomáš Eckschlager, 2^nd^ School of Medicine, Charles University, Prague, Czech Republic. The media were supplemented with 3% (L-15), or 10% (IMDM) newborn calf serum and a 100 U/mL penicillin, 100 μg/mL streptomycin (ref. number AAS-B/2; Capricorn Scientific) and 1% L-glutamine (ref. number 59202C; Sigma).

RAW 264.7 (mouse macrophage cell line; ATCC TIB-71) cells were cultivated DMEM medium supplemented with 5% FBS (ref. number FB-1001/ 100; Biosera), 1% Glutamine (ref. number MS0104101J, Biosera) and 1% antibiotics/antimycotics solution (ref. number AAS-B/2; Capricorn Scientific).

### Method details

#### DNA extraction and genotyping

DNA from blood from EU-TICK-BO cases were extracted using Promega Maxwell® 16 LEV Blood DNA Kits (Promega Corporation, WI 53711, USA) according to the manufacturers’ protocols. Extracted DNAs were quantified and quality checked using NanoDrop 2000TM (ThermoFisher Scientific, MA 02451, USA). Additionally, fluorescence-based quantification was performed using Quant-iT™ PicoGreen™ dsDNA Assay Kits from Invitrogen™ (Thermo Fisher Scientific, MA 02451, USA) on a LightCycler® 480 Instrument II (Roche Holding AG, 4070 Switzerland).

DNA aliquots of 50 ng/μl with a 260/280 ratio between 1.7 and 2.0 were sent to the Oxford Genomic Centre, Oxford, UK for genotyping using the Infinium© HTS assay on Global Screening Array bead-chips with the Multi-Disease drop in version 2.0 (GSA+MD v2.0; Illumina Inc., San Diego, California, USA) including nearly 760,000 genetic markers. Genotyped data from the German Heinz Nixdorf Recall Study (HNR), the Austrian Biomarkers of Personalized Medicine study (BPM) and Estonian Biobank (EBB) cohorts were generated by slightly different versions of bead-chips, namely, i) GSA+MD v1.0 in the HNR; ii) GSA v1.0 including custom markers in BPM; iii) GSA+MD v1.0 and/or v2.0 in EBB.

#### Genotype pre-processing and quality control

As TBE cases from the European Genetics Study of Tick-borne Encephalitis (EU-TICK-BO) and controls from the German Heinz Nixdorf Recall Study (HNR) and the Austrian Biomarkers of Personalized Medicine study (BPM) were genotyped separately, quality control (QC) was first performed independently for each dataset, followed by a second round of QC after genotype merging. Raw data from EU-TICK-BO cases and BPM controls were pre-processed using Illumina GenomeStudio2, excluding samples with call rate < 0.95 and variants with genotyping rate < 0.95 or minor allele frequency (MAF) < 0.001. We used plink2 and plink1.9 for QC. Whenever the option was available in both plink2^45^ and plink1.9^52^, we used plink2 to perform the analysis. For sample-based QC, we removed samples with sex discrepancies, heterozygosity rate±5 SD away from the sample mean, samples with >2% of missing data, first and second-degree relatives or duplicate samples based on KING’s kinship estimator (Kinship>0.08838835) as well as Identity by Descend (PI_HAT>0.185). In case of related/duplicate samples, one of the pairs of related samples with high genotype missing rate was removed. For variant based QC, we removed variants with genotyping rate of <98%, minor allele frequency (MAF) <0.01, failing hardy Weinberg equilibrium P value ( P<1×10^-6^ for controls and P<1×10^-10^ for cases), located on sex chromosomes and duplicate SNPs. Finally, each cohort was checked for population outlier using principal component analysis (PCA) and they were removed by visual inspection.

After the first stage of QC, each cohort was realigned to 1000 Genome reference dataset using genotype harmoniser.^53^ During this stage, ambiguous A/T G/C with 40 to 60% frequency were also removed. Finally, EU-TICK-BO, HNR, and BMP cohorts were merged on shared common SNPs to form EU-TICK-BO-plus cohorts (N=432,077).

As a second round of QC post-merging, we repeated the sample QC removing samples that failed based on missingness, heterozygosity and relatedness. For variant QC, we removed variants with missingness of >2%, HWE <0.001, MAF <0.01, and additionally based on differential missingness ( P<1×10^-5^).

The EBB cohort only underwent the one round of QC as cases and controls were genotyped simultaneously. In the EBB cohort, there were 447,663 SNPs remained after QC.

After these QC steps, the merged EU-TICK-BO-plus and the EBB cohorts underwent a final ancestry check using PCA with 1000 genomes data to detect European ancestry outliers using bigsnpr^47^ and Plink2.^45^

At this stage we performed GWAS for susceptibility to TBE using generalised linear mixed models implemented in fastGWA-GLMM^26^ on the EU-TICK-BO-plus and EBB cohorts to check whether the QC steps for control of genomic inflation were optimal. Genomic inflation was 1.025 in the EU-TICK-BO-plus cohort and 1.007 in the EBB.

#### Imputation and post-imputation QC

Before uploading the data to the Michigan Imputation Server^46^ for imputation, additional QC steps were performed using QC toolbox (https://www.chg.ox.ac.uk/∼wrayner/tools/).

We used Eagle 2.4^54^ for phasing and imputed to the Haplotype Reference Consortium (HRC) reference panel using minimac4.^55^ The EU-TICK-BO-plus and EBB cohorts were imputed separately to the GRCh37 genotype assembly. We downloaded all imputed variants with R^2^ >0.3. After imputation we removed variants with imputation quality R^2^ <0.8, MAF <0.01 and HWE P <1×10^-6^. Finally, 7,007,551 SNPs from the EU-TICK-BO-plus cohort and 7,247,027 from the EBB cohort were available for analyses.

#### RNA sequencing

A subgroup of prospectively recruited participants of the EU-TICK-BO cohort had the collection of PAXgene tubes (N = 23). RNA was isolated from peripheral blood samples stored in PAXgene tubes using the QIAsymphony PAXgene Blood RNA Kit on the QIAsymphony system (QIAGEN N.V., Hilden, Germany). Extracted RNA with an RNA Integrity Number (RIN) >7 was further processed using the NEBNext® Globin & rRNA Depletion Kit (New England Biolabs, MA, US) for globin and ribosomal RNA removal. RNA libraries were then prepared with the NEBNext® Ultra™ II Directional RNA Library Prep Kit for Illumina® (New England Biolabs, MA, US). Sequencing was performed on a NovaSeq 6000 platform (Illumina, Inc, San Diego, California, US), generating 100 base pair paired-end reads.

The sequencing reads were aligned to the Ensembl gene reference genome Homo sapiens GRCh38 using the STAR aligner,^48^ and featureCounts^49^ was used to quantify gene expression in the Galaxy software suite. The resulting count data were pre-processed and normalised using the variance stabilising transformation in DESeq2 (V1.34.0).^50^

#### *In vitro* exploration

##### Toxicity test

The benzamil (pharmacological inhibitor of ABCG1^56^; Sigma-Aldrich) cytotoxicity was determined in terms of cell viability using the Cell Counting Kit-8 (CCK-8; Dojindo Molecular Technologies, Munich, Germany) according to manufacturer’s instructions. The UKF-NB-4 cells were seeded in 96-well plates (approximately 3 x 10^4^ cells per well) and incubated for 24 h at 37 °C to form a confluent monolayer. The medium was then aspirated from the wells and replaced with 200 μL of fresh medium containing 0, 6.25, 12.5, 25 or 50 μM of benzamil. After 24 h and 48 h incubation, the medium in each well of the plates was replaced by fresh medium containing CCK-8 solution in appropriate concentration. The plates were incubated for 1 h in the incubator. The absorbance was measured at 450 nm using a microplate reader.

Confluent monolayers of UKF-NB-4 cells cultured in 96-well plates were infected with the TBEV Hypr strain (provided by the Collection of Arboviruses, Institute of Parasitology, Biology Centre of the Czech Academy of Sciences, Ceske Budejovice, Czech Republic) at a multiplicity of infection (MOI) of 0.1. The cells were treated with 0, 6.25 μM, or 12.5 μM benzamil and 10 μM cholesterol-water soluble (both from Sigma Aldrich). In the pre-treatment variant, UKF-NB-4 cells were exposed to benzamil 24 hours prior to infection at the same concentrations.

At 24 hours or 48 hours post-infection, the supernatant was collected, and TBEV titres were determined using plaque assays, following a modified protocol as previously described^57^ Briefly, 10-fold dilutions of TBEV were prepared in 24-well tissue culture plates, and 1 × 10^5^ PS cells per well were added in suspension. After 4 hours of incubation at 37°C, the suspension was overlaid with 1.5% (w/v) carboxymethylcellulose in L-15 medium. Plates were incubated at 37°C for 5 days, washed with phosphate-buffered saline (PBS), and the monolayers were stained with naphthalene black. Virus titres were expressed as plaque-forming units per millilitre (PFU/ml).

Statistical analysis of the data was performed using the nonparametric Kruskal-Wallis test, followed by Dunn’s multiple comparison test, in GraphPad Prism (version 7.04). A p-value < 0.05 was considered statistically significant.

The effect of benzamil on TBEV replication was also evaluated using a recombinant TBEV Hypr strain expressing the mCherry reporter protein.^58^ UKF-NB-4 cells were cultured in 96-well plates and treated with benzamil (0 to 12.5 μM) as described above. Cells were then infected with the recombinant reporter TBEV at a MOI of 0.1. At 24- and 48-hours post-infection, cell nuclei were visualized using Hoechst 33342 dye (Invitrogen™) following the manufacturer’s protocol, and mCherry expression was analysed via fluorescence microscopy.mImages were captured using an Olympus IX81 epifluorescence microscope equipped with a Hammamatsu OrcaR2 camera and controlled by Xcellence software. Image processing and analysis were conducted using ImageJ/Fiji software.^59^^,^^60^

#### Effect of *ABCG1* knock-down on TBEV growth

Silencer Select Pre-designed siRNA targeting *ABCG1* (s61797, Ambion®, Life Technologies, USA) and Silencer™ Select Negative Control No. 1 siRNA (catalog number 4390843, Thermo Fisher Scientific) were procured from Thermo Fisher Scientific (Waltham, MA, USA). Transfection was conducted on adherent cells using the Lipofectamine™ RNAiMAX Transfection Reagent (Thermo Fisher Scientific, Waltham, MA, USA) according to the manufacturer’s protocol.

One day before transfection, 1 × 10^5^ RAW 264.7 cells (mouse macrophage cell line) were seeded into a 24-well plate. The following day, for each well, 6 pmol of siRNA was diluted in Opti-MEM™ Reduced Serum Medium (Gibco, Life Technologies, USA) without serum and gently mixed. Separately, 1 μL of Lipofectamine RNAiMAX was diluted in 50 μL of Opti-MEM™ Reduced Serum Medium and gently mixed. The siRNA-Lipofectamine RNAiMAX/Opti-MEM complex was incubated for 20 minutes at room temperature before being added to each well to achieve a final RNA concentration of 10 nM. The cells were incubated at 37°C in a CO_2_ incubator.

Successful downregulation of *ABCG1* expression was verified using RT-qPCR every 24 hours up to 7 days post-transfection (dpt); no changes in ABCG1 expression were detected in control cells transfected with the negative control siRNA. Total RNA was isolated from the cells using the QIAamp Viral RNA Mini Kit (QIAGEN). RT-qPCR was conducted using the Luna® Probe One-Step RT-qPCR Kit (New England Biolabs) and TaqMan™ Gene Expression Assays (Applied Biosystems, Thermo Fisher Scientific). Amplification was performed on a qTOWER^3^G thermocycler (Analytik Jena, Jena, Germany).

TBEV was applied at 48 hours post-transfection (hpt) at aN MOI of 0.1. Medium was collected every 24 hours up to 5 days post-treatment (dpt), and TBEV titers were determined via plaque assays.

Statistical analysis of the data was performed using the nonparametric Kruskal-Wallis test, followed by Dunn’s multiple comparison test, in GraphPad Prism (version 7.04). A p-value < 0.05 was considered statistically significant.

### Quantification and statistical analysis

#### Genome-wide association study and meta-analysis

Association analysis was performed using generalized linear mixed models implemented in fastGWA-GLMM^26^ using genotype dosage data. The analysis in the EU-TICK-BO*-*plus cohort was adjusted for five PCs and sex, while in the EBB cohort, it was adjusted for six PCs and sex to minimise genetic inflation. Meta-analysis was performed using inverse variance weighted methods in METAL.^61^ We have used P<1×10^-5^ as a suggestive-significance and P<5×10^-8^ as a genome-wide significance.

#### Post-GWAS analysis

Singleton variants with P<1×10^-5^ were removed from GWAS results, and the remaining variants were clumped using r^2^ threshold of 0·3 to identify independent loci. Gene-based analyses were performed using the MAGMA^27^ software implemented within FUMA,^28^ where SNPs were mapped to 18,622 protein-coding genes. To capture regulatory variants, we included variants within 10 kilobases of the 3′ and 5′ untranslated regions of each gene. The analysis utilised a multiple linear principal components regression model, with gene-wide significance defined as P<2·68×10^-6^. Additionally, variants were annotated using Haploreg.^31^

#### *ABCG1* expression analysis

Normalised expression counts for *ABCG1* were extracted from the count matrix obtained from DESeq2. Differential expression of *ABCG1* between homozygous and heterozygous individuals was assessed using a generalised linear model, adjusting for sex as a covariate.

#### Statistical analysis

Statistical analysis for *in vitro* experiments was performed using the nonparametric Kruskal-Wallis test, followed by Dunn’s multiple comparison test, in GraphPad Prism (version 7.04). A p-value < 0.05 was considered statistically significant. The differences in *ABCG1* gene expression between people with and without the rs3787986 variant was tested using a generalised linear model adjusted for sex.

In the post-hoc power calculation, our sample size of 1,600 cases and 9,699 controls had ∼80% power to detect OR ≥ 1.33 at MAF=0.23 using two-sided α=5×10-^8^. Power calculation was done using R package “genpwr” version 1.0.4.

#### Plotting

Quantile-Quantile (QQ) and Manhattan plots were generated using the topR^62^ package, while LocusZoom plots were created with the LocusZoom.^63^ Other analyses were performed in R version 4.4 using the ggplot2^64^ library within Rstudio (2022.02.2+485 “Prairie Trillium”).^65^

## Supplemental information

Document S1. Figures S1–S12 and Table S1

Table S2. Meta-analysis of GWAS of susceptibility to TBE (All suggestive SNPs)

Table S3. eQTL analysis results from the eQTLGen database for GWAS suggestive variants in the ABCG1 region

Table S4. Association results for severity analysis
