# Supplementary material for: Genome-wide association study identifies ABCG1 as a susceptibility locus for tick-borne encephalitis
Source: iScience. 2025 Nov 12;28(12):114017. doi: 10.1016/j.isci.2025.114017 (PMC12686730; doi:10.1016/j.isci.2025.114017)
Supplement: Document S1. Figures S1–S12 and Table S1 [file mmc1.pdf]

iScience, Volume *28*

Supplemental information

Genome-wide association study identifies *ABCG1* as a susceptibility locus for tick-borne encephalitis

Piyush G. Gampawar, Manfred G. Sagmeister, Daniel Růžek, Nina A. Schweintzger, Edith Hofer, Benno Kohlmaier, Vendula Švendová, Petra Bogovič, Joanna M. Zajkowska, Lenka Krbková, Věra Štruncová, Auksė Mickienė, Daniela S. Kohlfürst, Astrid Sonnleitner, Andrea Fořtová, Michaela Berankova, Martina Pychova, Dace Zavadska, Neneh Sallah, Alexander Pichler, Dalibor Sedláček, Aleš Chrdle, Christoph Haudum, Barbara Obermayer-Pietsch, Per Hoffmann, Markus M. Nöthen, Mari-Liis Tammesoo, Andres Metspalu, Petr Husa, Karin Stiasny, Alexander Binder, Andrea Berghold, Franc Strle, Martin L. Hibberd, Werner Zenz, and the EU-TICK-BO study group

Supplementary Figures

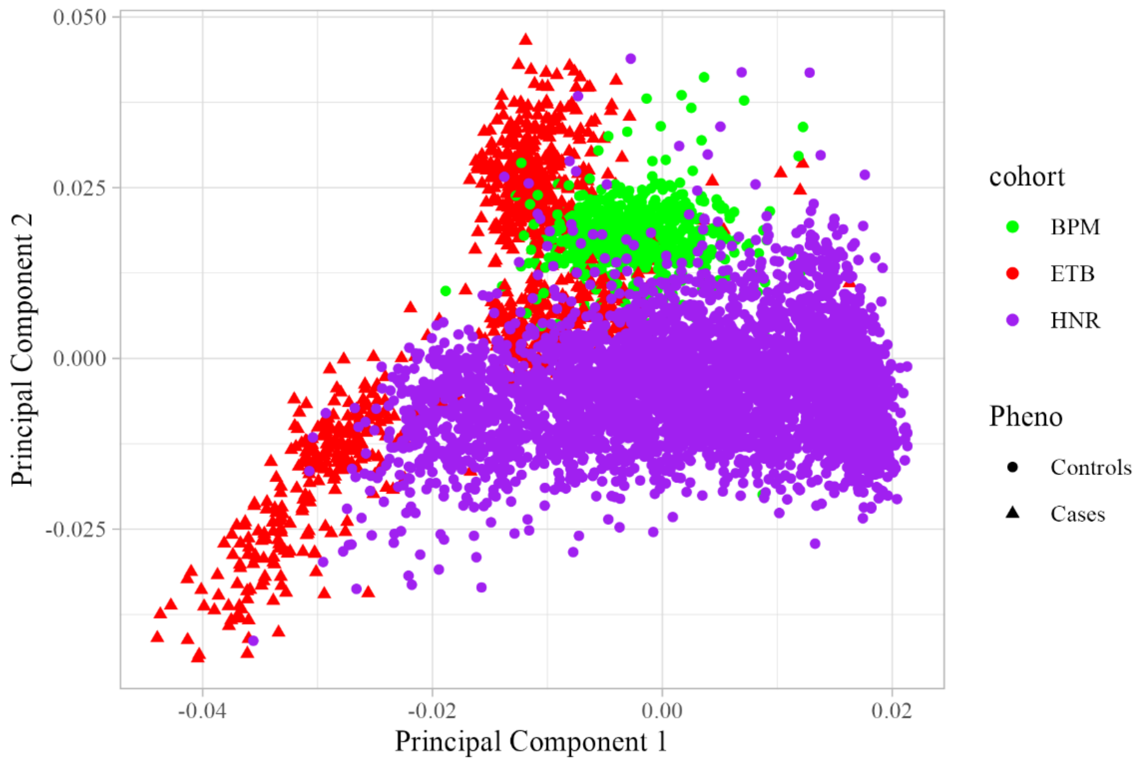

Figure S1*: Principal component analyses of TBE cases and controls in EU-TICK-BO-plus cohort.* Principal components were generated using the PCA method implemented in PLINK2 with an LD-pruned subset of SNPs. After QC, PCA revealed no population outliers. ETB: EU-TICK-BO cohort, BPM: Biomarkers of Personalised Medicine cohort, HNR: Heinz Nixdorf Recall Study cohort

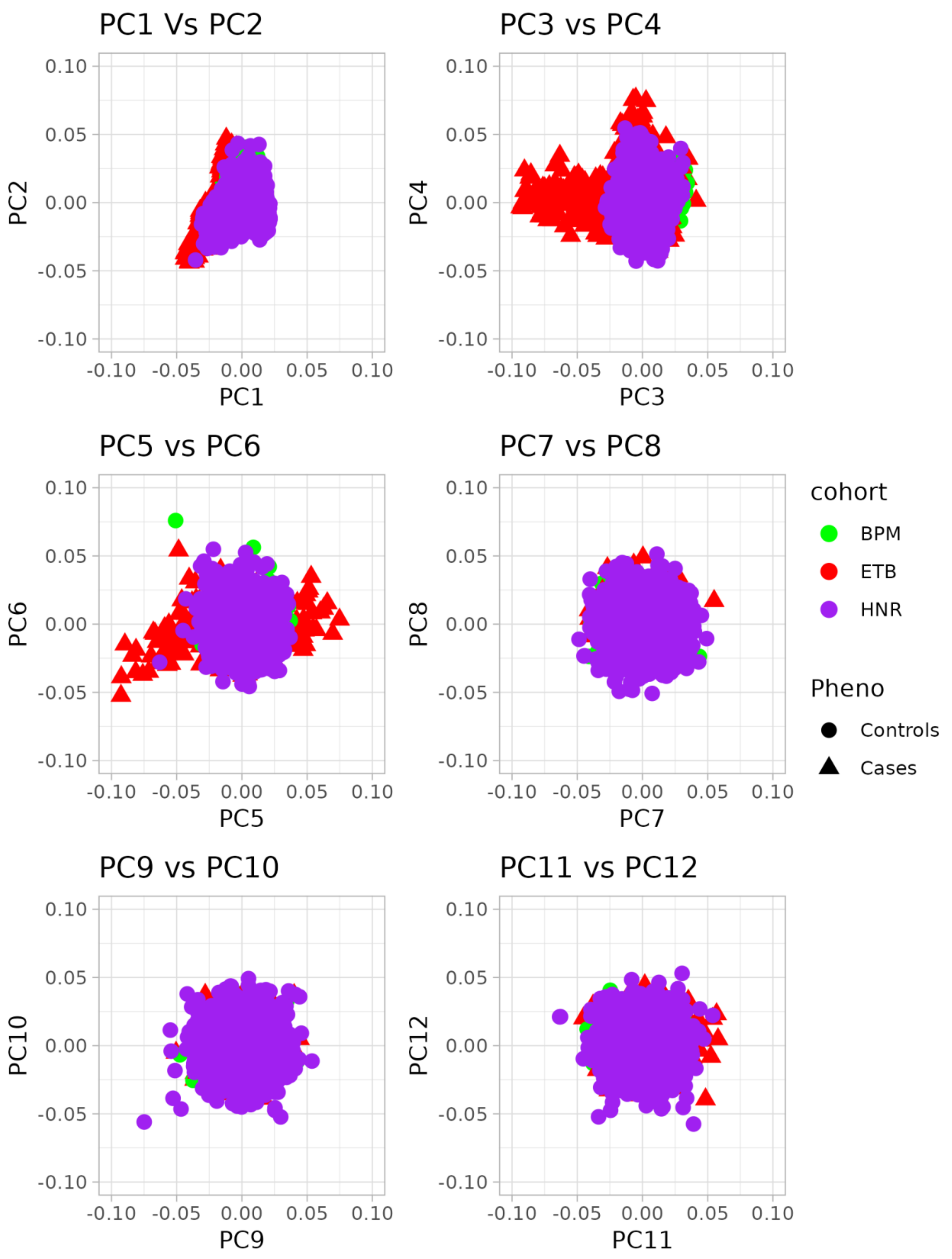

Figure S2: ***First 12 Principal component of TBE cases and controls in EU-TICK-BO-plus cohort after quality control.*** Principal components were generated using the PCA method implemented in PLINK2 with an LD-pruned subset of SNPs. After QC, PCA revealed no population outliers. ETB: EU-TICK-BO cohort, BPM: Biomarkers of Personalised Medicine cohort, HNR: Heinz Nixdorf Recall Study cohort

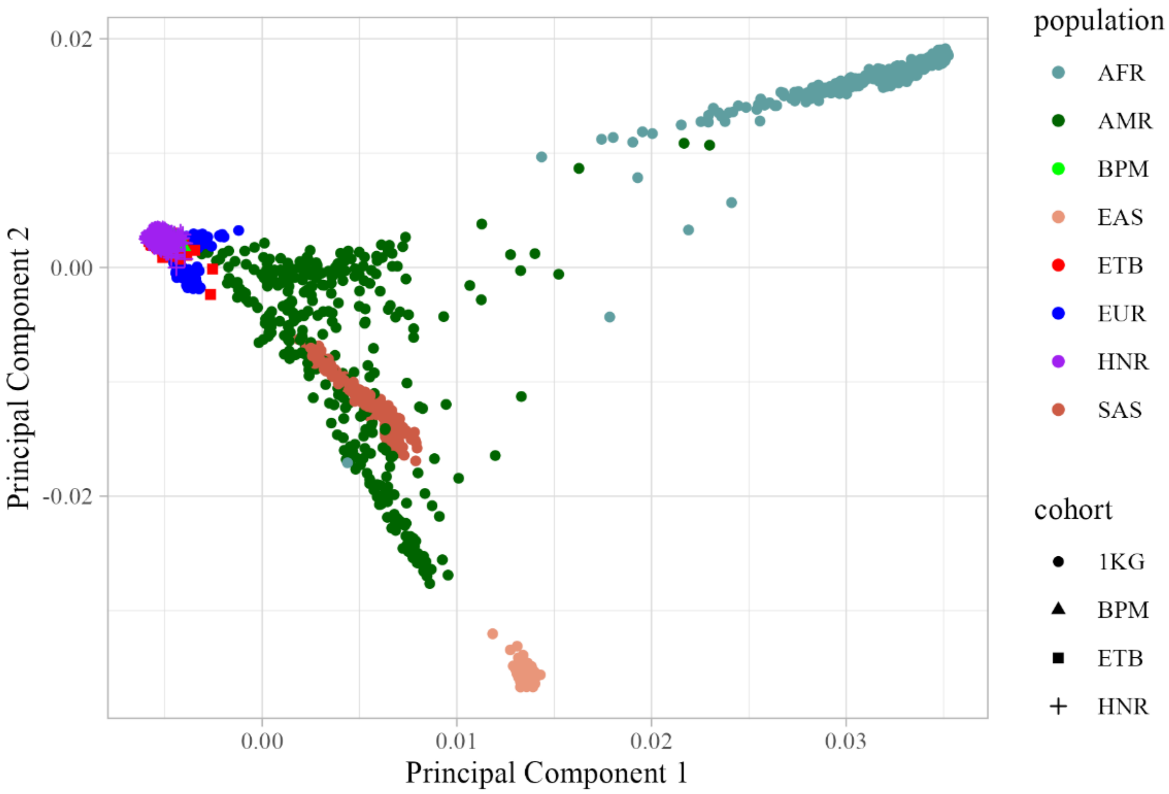

Figure S3*: Principal component analyses of TBE cases and controls in EU-TICK-BO-plus cohort projected on samples from 1000 genome.* Cases and controls from EU-TICK-BO-plus cohorts were projected along with samples from 1000 genome project with known ancestry. Principal components were generated using the PCA method implemented in PLINK2 with an LD-pruned subset of SNPs. Ancestry outliers, i.e., ancestry not matching European populations, were removed. After QC, PCA revealed no non-European ancestry outliers.

AFR: Africans, AMR: Americans, EAS: East Asians, SAS: South Asians, EUR: Europeans, ETB: EU-TICK-BO cohort, BPM: Biomarkers of Personalised Medicine cohort, HNR: Heinz Nixdorf Recall Study cohort

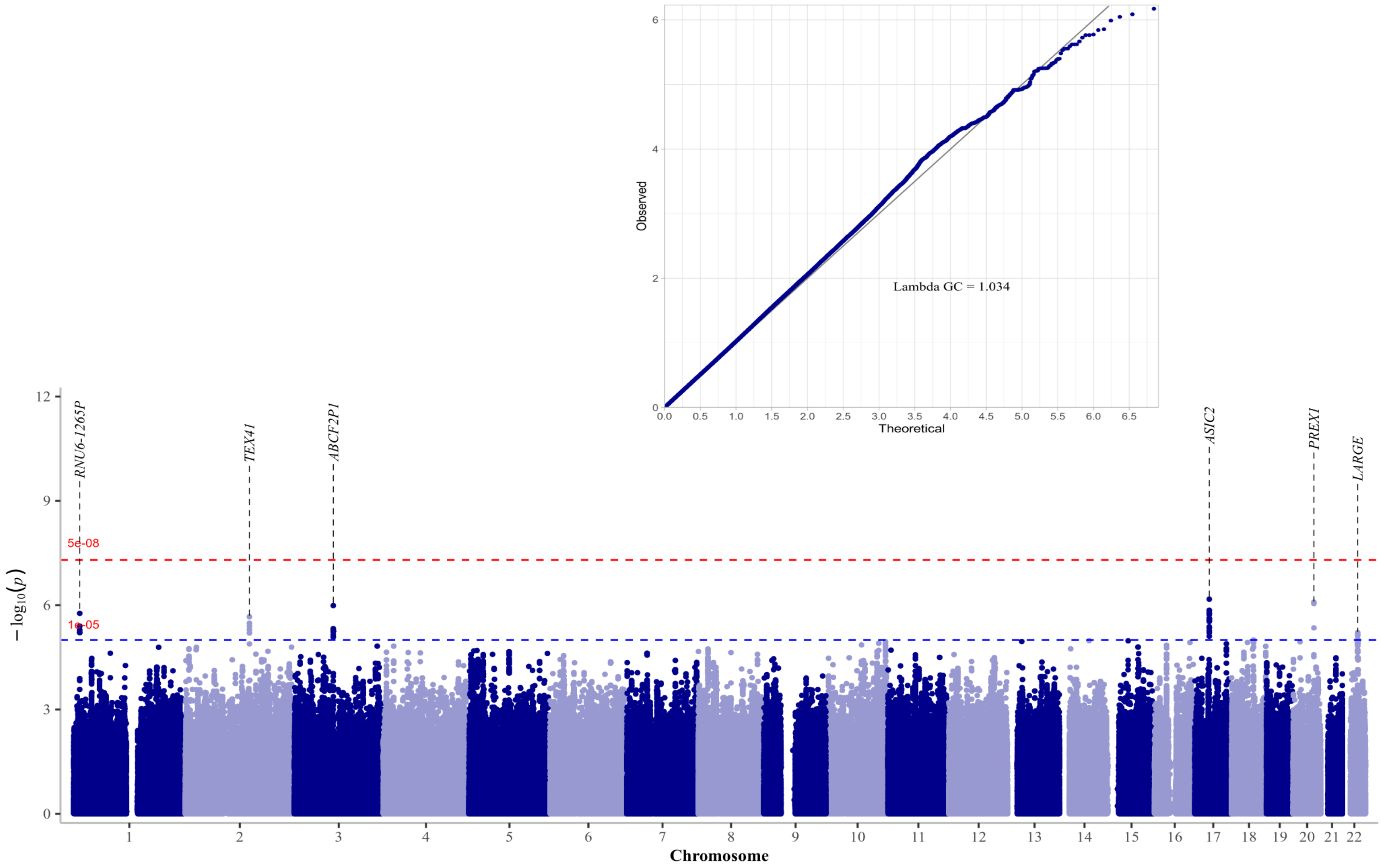

Figure S4: Quantile-Quantile and Manhattan plots of EU-TICK-BO-plus cohort Analysis for Susceptibility to TBE.

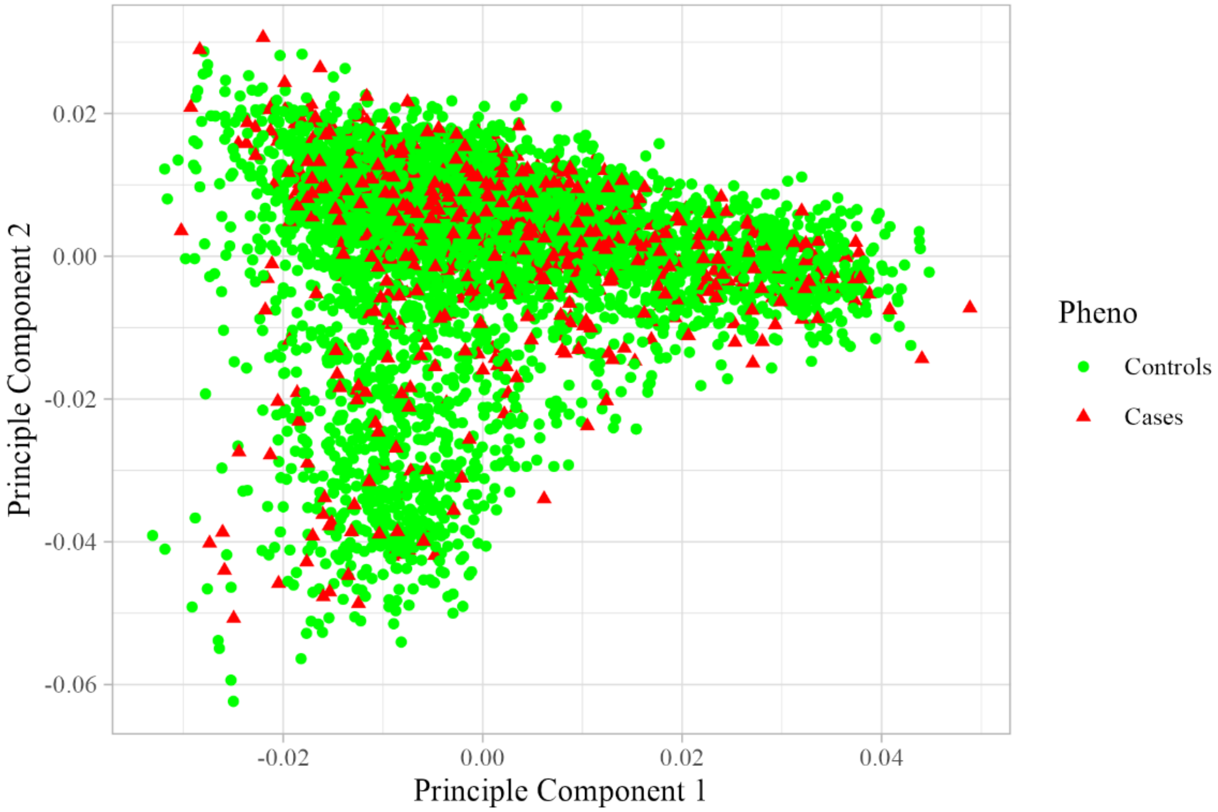

Figure S5*: Principal component analyses of TBE cases and controls in Estonian Biobank cohort.* Principal components were generated using the PCA method implemented in PLINK2 with an LD-pruned subset of SNPs. After QC, PCA revealed no population outliers. After QC, PCA revealed no population outliers

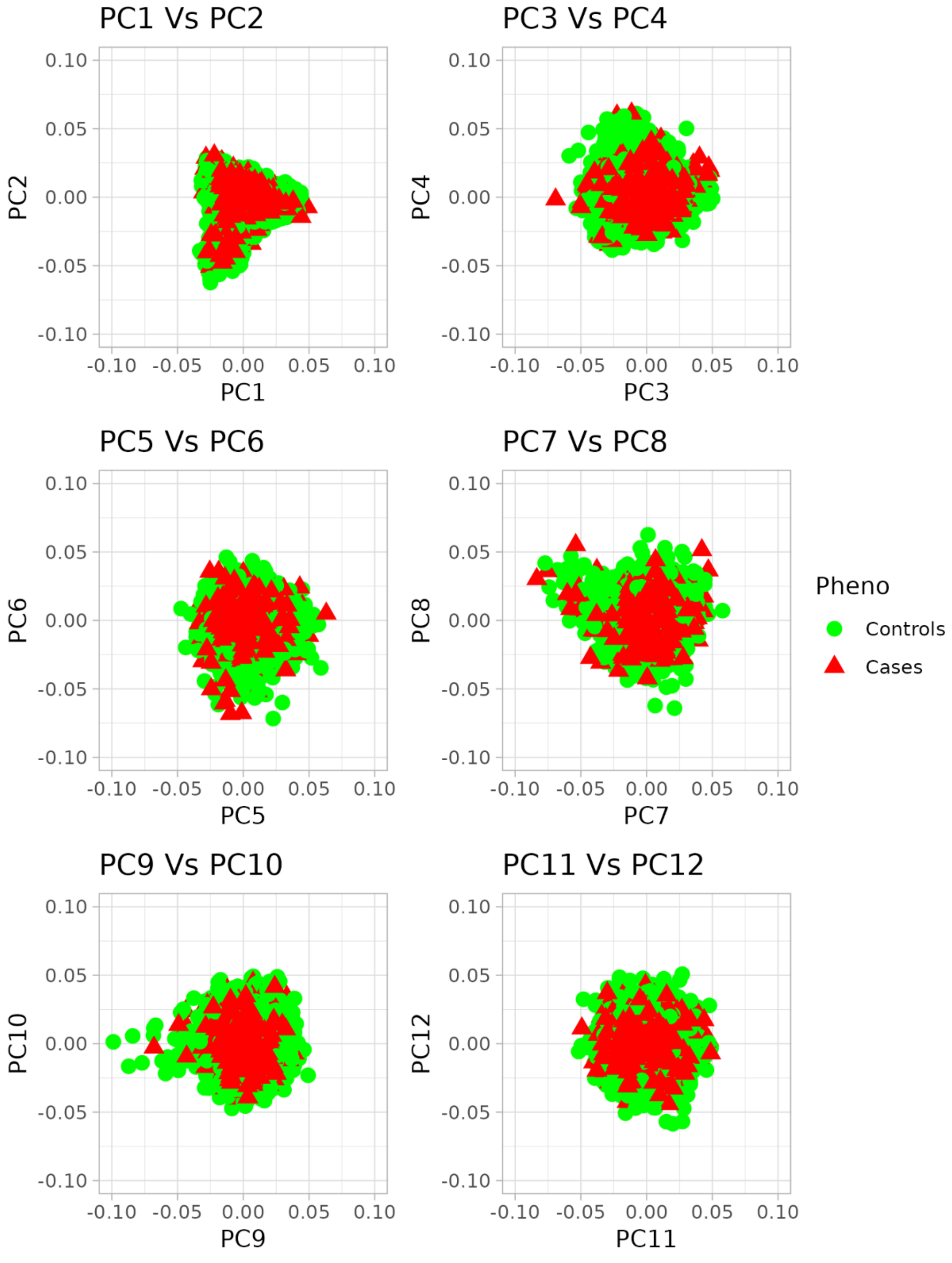

Figure S6*: of TBE cases and controls in Estonian Biobank cohort after quality control.* Principal components were generated using the PCA method implemented in PLINK 2 with an LD-pruned subset of SNPs. After QC, PCA revealed no population outliers. After QC, PCA revealed no population outliers

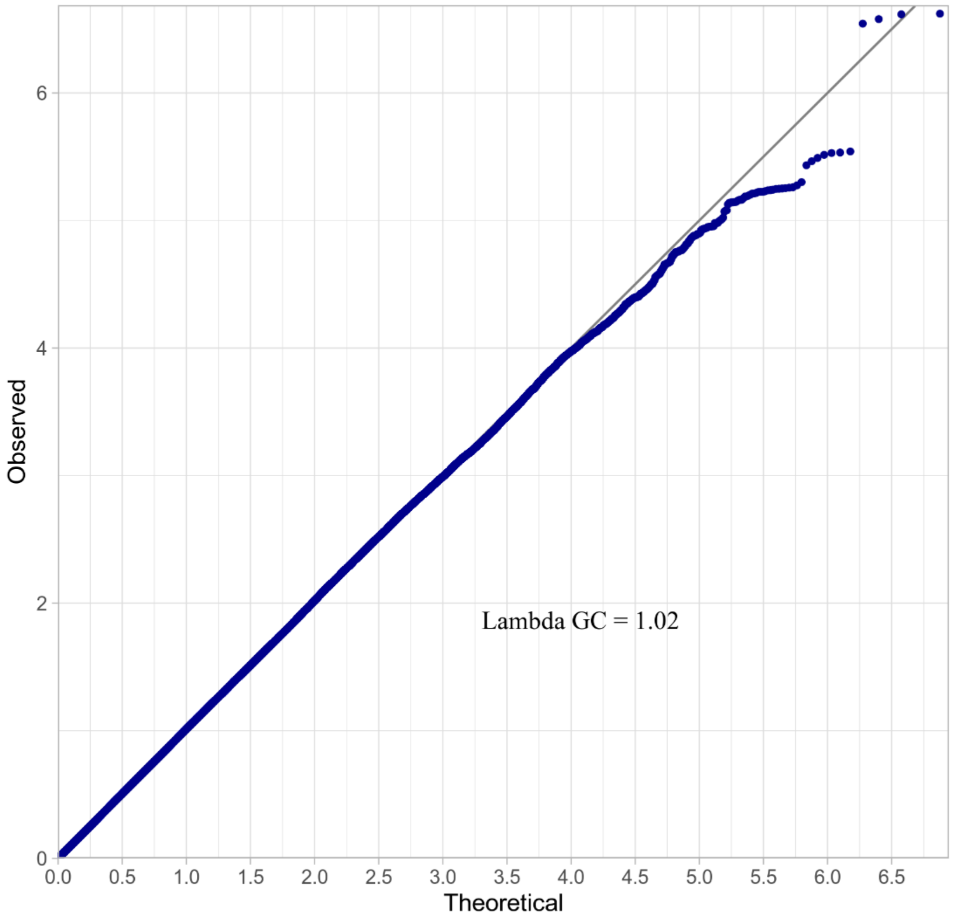

Figure S8*: Quantile-Quantile Plot of Meta-Analysis for Susceptibility to TBE*

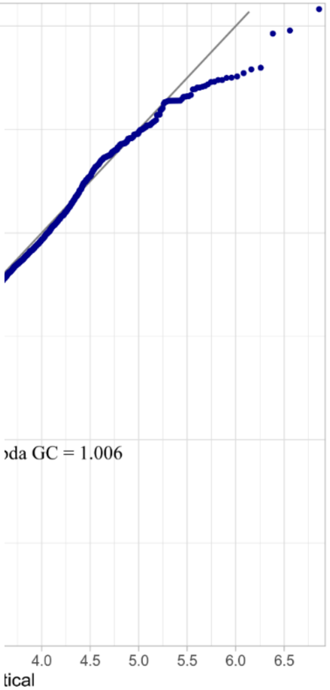

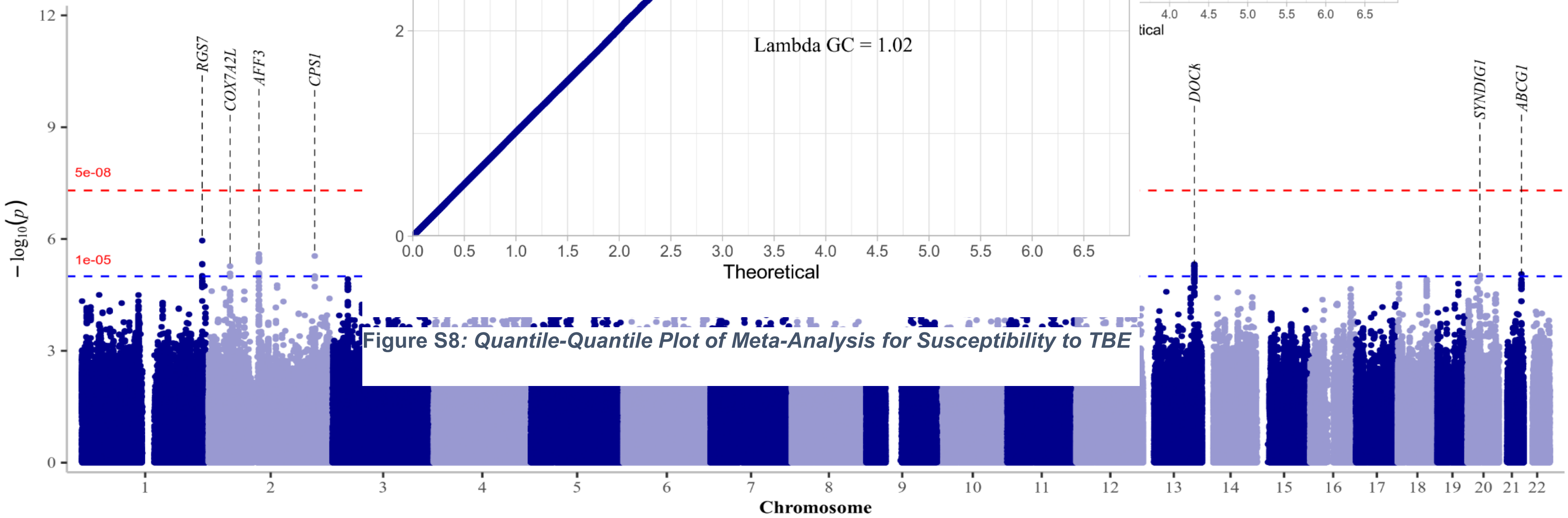

Figure S7*: Quantile-Quantile and Manhattan plots of EBB cohort Analysis for Susceptibility to TBE.*

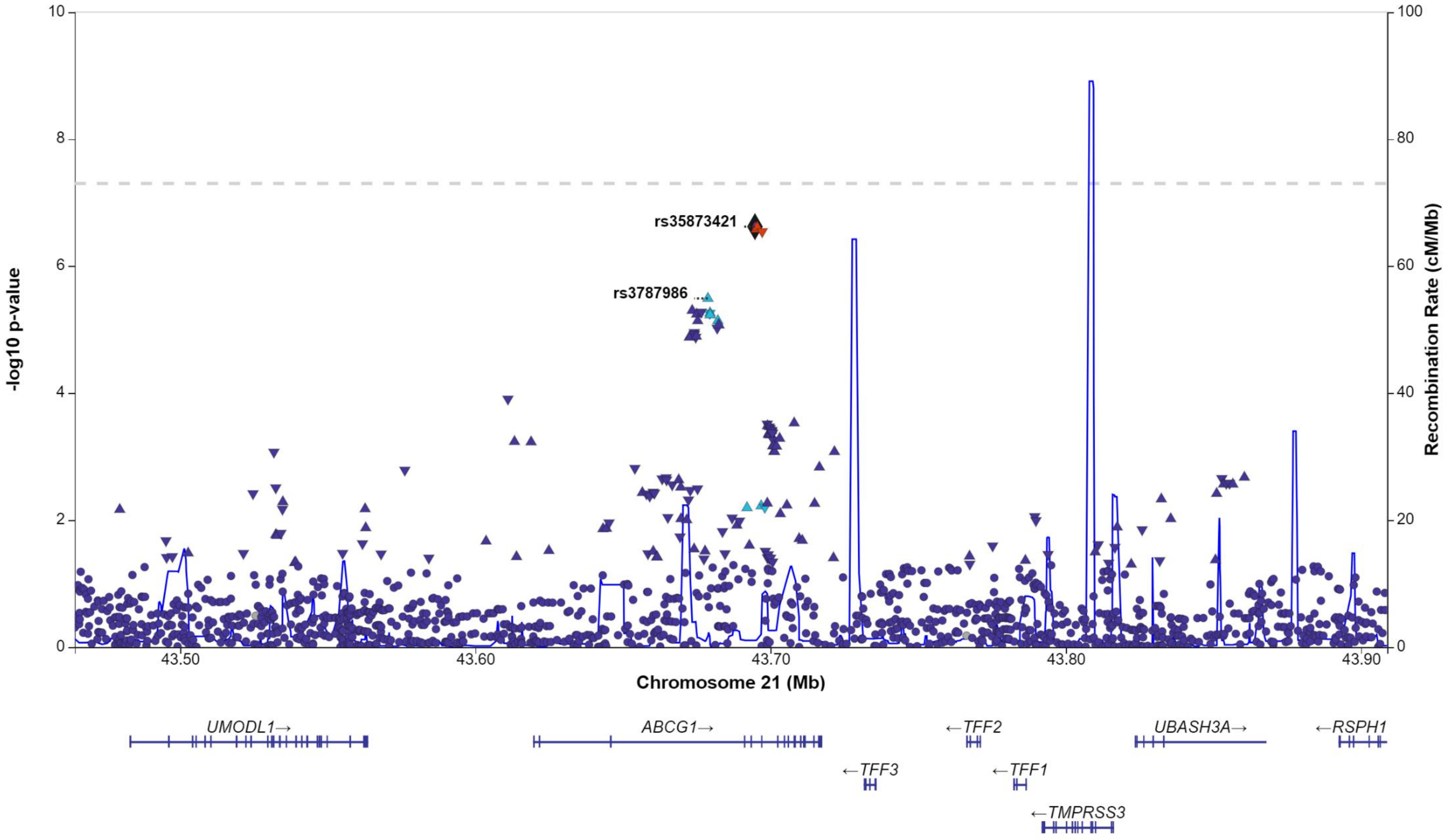

Figure S9: LocusZoom Plot of Meta-Analysis for Susceptibility to TBE Highlighting the Lead SNP in the ABCG1 Region.

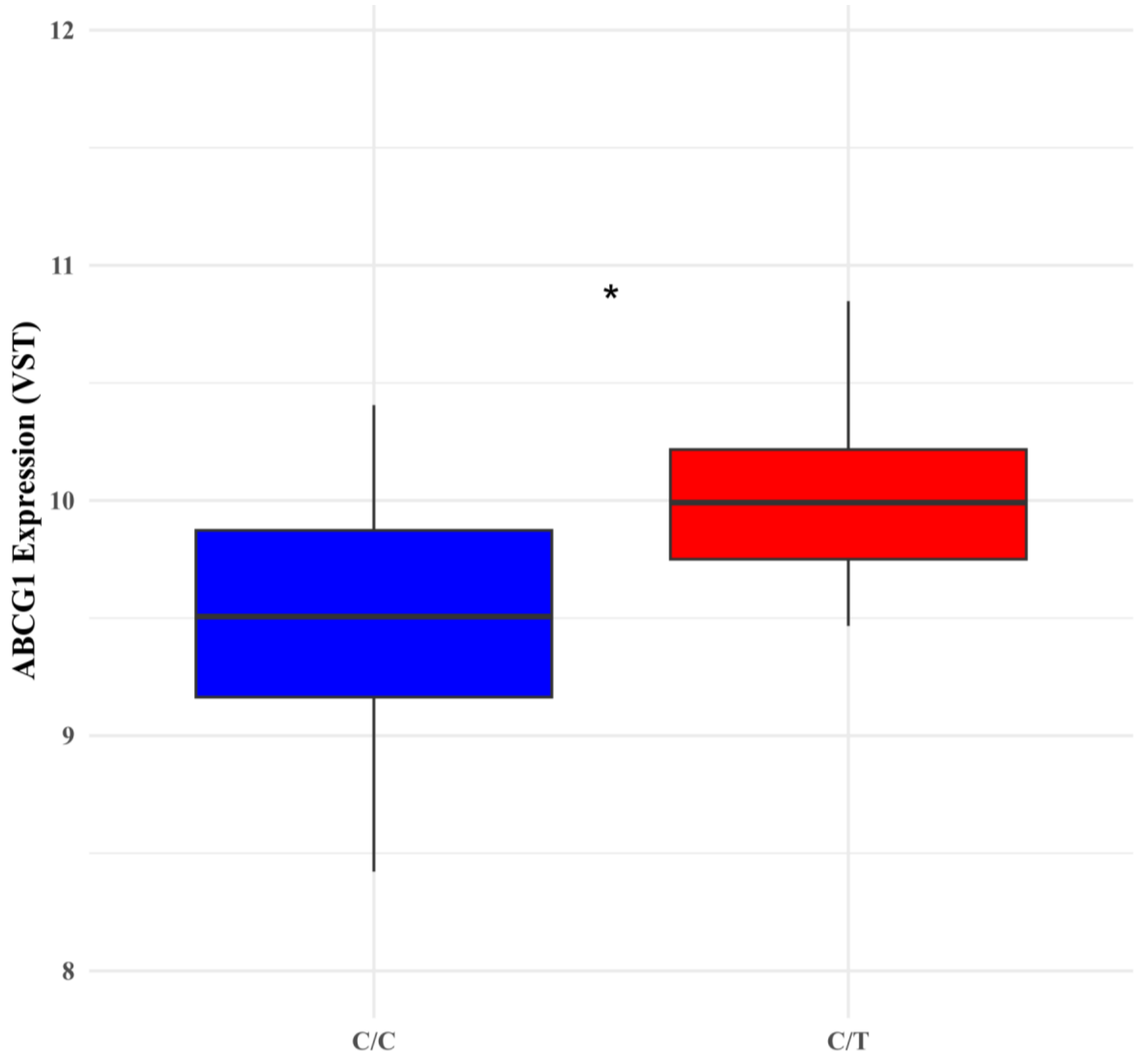

Figure S10 Boxplot illustrating differences in ABCG1 expression among individuals with and without SNP rs3787986. RNA expression from peripheral blood was measured using RNA sequencing, and expression counts were normalised using vst transformation in DESeq2. The comparison between individuals homozygous for the reference allele and those heterozygous for the T allele was performed using a generalised linear model, adjusting for sex. Y-axis is normalised *ABCG1* expression. Analysis was performed using a generalised linear model adjusted for sex.

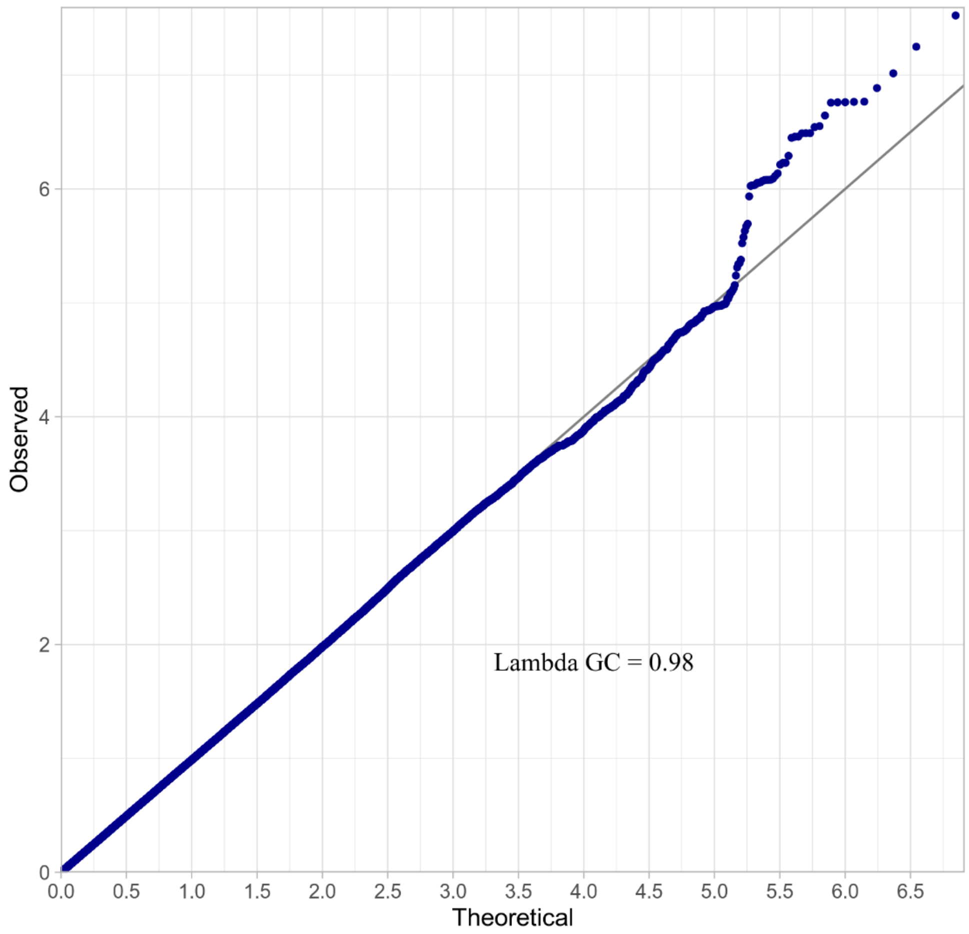

Figure S11: Quantile-Quantile Plot of Meta-Analysis for Susceptibility to severe forms of TBE

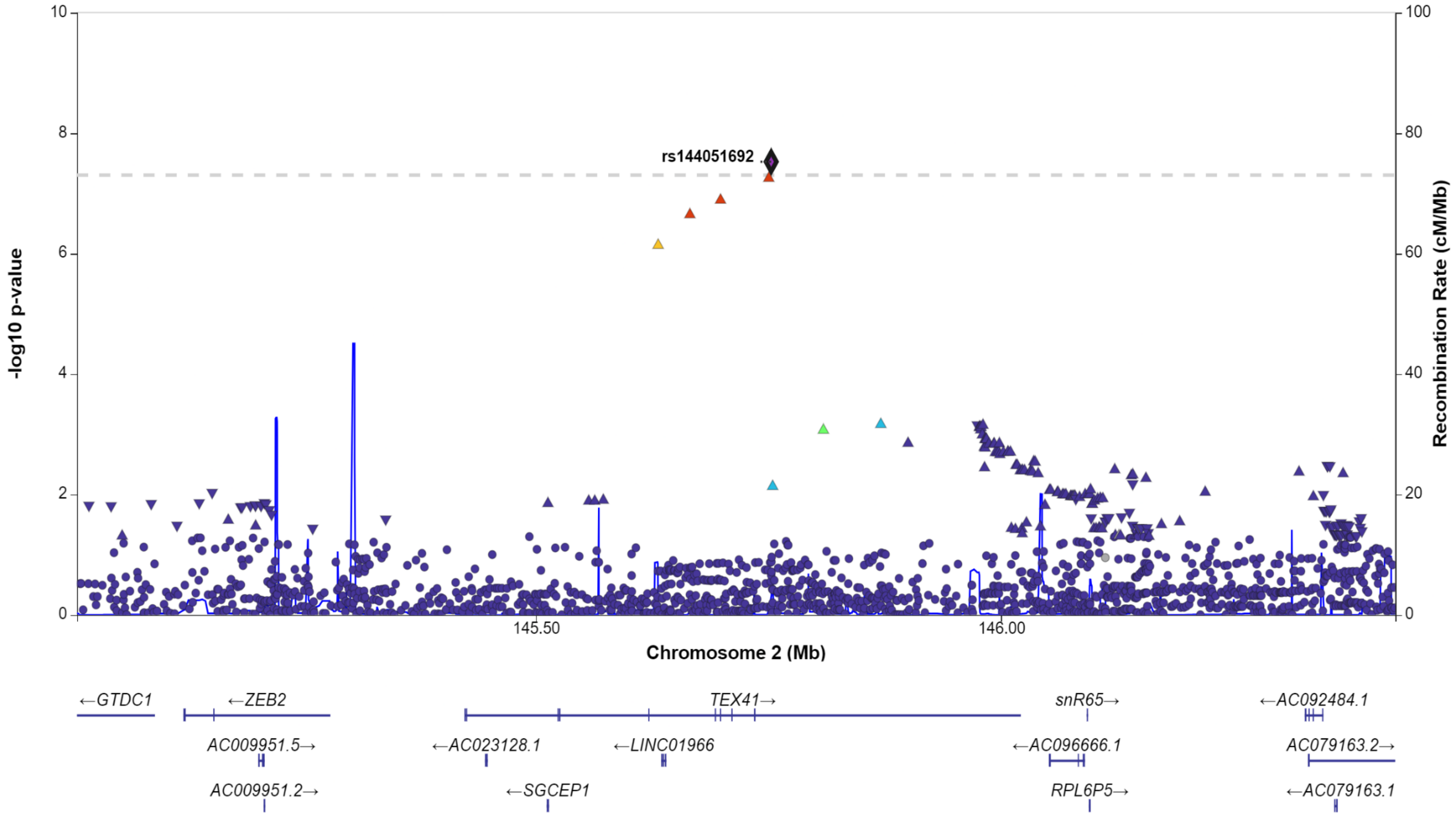

Figure S12: LocusZoom Plot of GWAS for Susceptibility to the Severe forms of TBE Highlighting the Lead SNP.

Supplementary Tables

Table S1: Recruitment of TBE cases in the EU-TICK-BO cohort.

**Country**	**Number of TBE cases**
Austria	27
Czechia	233
Latvia	17
Lithuania	92
Poland	161
Slovenia	408
**Total**	**938**
